# Co-expression module analysis reveals high expression homogeneity for both coding and non-coding genes in sepsis

**DOI:** 10.1186/s12864-023-09460-9

**Published:** 2023-07-24

**Authors:** Xiaojun Liu, Chengying Hong, Yichun Jiang, Wei Li, Youlian Chen, Yonghui Ma, Pengfei Zhao, Tiyuan Li, Huaisheng Chen, Xueyan Liu, Lixin Cheng

**Affiliations:** Department of Critical Care, Shenzhen People’s Hospital, First Affiliated Hospital of Southern University of Science and Technology, Second Clinical Medicine College of Jinan University, Shenzhen, 518020 China

**Keywords:** Co-expression network, Gene module, Sepsis, Non-coding RNA

## Abstract

Sepsis is a life-threatening condition characterized by a harmful host response to infection with organ dysfunction. Annually about 20 million people are dead owing to sepsis and its mortality rates is as high as 20%. However, no studies have been carried out to investigate sepsis from the system biology point of view, as previous research predominantly focused on individual genes without considering their interactions and associations. Here, we conducted a comprehensive exploration of genome-wide expression alterations in both mRNAs and long non-coding RNAs (lncRNAs) in sepsis, using six microarray datasets. Co-expression networks were conducted to identify mRNA and lncRNA modules, respectively. Comparing these sepsis modules with normal modules, we observed a homogeneous expression pattern within the mRNA/lncRNA members, with the majority of them displaying consistent expression direction. Moreover, we identified consistent modules across diverse datasets, consisting of 20 common mRNA members and two lncRNAs, namely CHRM3-AS2 and PRKCQ-AS1, which are potential regulators of sepsis. Our results reveal that the up-regulated common mRNAs are mainly involved in the processes of neutrophil mediated immunity, while the down-regulated mRNAs and lncRNAs are significantly overrepresented in T-cell mediated immunity functions. This study sheds light on the co-expression patterns of mRNAs and lncRNAs in sepsis, providing a novel perspective and insight into the sepsis transcriptome, which may facilitate the exploration of candidate therapeutic targets and molecular biomarkers for sepsis.

**Supplementary Information:**

The online version contains supplementary material available at 10.1186/s12864-023-09460-9.

## Introduction

Sepsis is life-threatening organ dysfunction caused by a dysregulated host response to infection. Sepsis and septic shock are major healthcare problems affecting about 20 million of people worldwide each year with the mortality as high as 20% [1]. Despite its impact, effective treatments for sepsis remain elusive [2, 3]. Recent advancements in high-throughput technologies, coupled with the availability of a vast number of publicly available data and sophisticated algorithms, have opened up possibilities for mining disease-related genes [3–8]. However, previous studies have primarily focused on individual gene functions in sepsis, disregarding the fact that genes tend to work together to carry out cellular processes and regulate signaling pathways [9–11]. From a system biology perspective, disease-related genes are frequently co-expressed across a set of samples, indicating their collaborative role rather than functioning independently [12–16].

Moreover, while numerous studies have explored the expression patterns of coding genes in sepsis, the comprehensive assessment of long non-coding RNAs (lncRNAs) and their potential biological functions in sepsis remains largely unexplored [17–19]. lncRNAs are non-protein-coding transcripts exceeding 200 nucleotides in length and have been discovered to function as regulators involved in various biological processes [20–22]. Emerging evidence suggests that lncRNAs play significant roles in several immunological processes [20, 23]. However, to date, no systematic studies have investigated the importance of lncRNAs in sepsis responses.

As large-scale network data become pervasive in biological omics studies, algorithms for detection of molecular modules from networks are of critical importance. Although dozens of algorithms have been developed for module identification, including MCODE, ClusterONE, SMILE, LTOP, WGCNA, etc., no single type of approach is inherently superior [13, 14, 24]. Molecular Complex Detection (MCODE) detects densely interconnected clusters from protein-protein interaction (PPI) networks that may represent protein complexes. It uses vertex weighting (a form of the clustering coefficient) to extend clusters from an initial vertex of high local weight by iteratively adding neighboring vertices with similar weights. Clustering with Overlapping Neighborhood Expansion (ClusterONE) is a graph clustering algorithm that is able to handle weighted graphs and readily generates overlapping clusters [25]. It is especially useful for detecting protein complexes in PPI networks with associated confidence values. ClusterONE takes into account the confidence values and readily generates overlapping clusters, showing decent correspondence with the MIPS catalogue of protein complexes in complex prediction. Cheng et al. proposed subcellular module identification with localization expansion (SMILE) to detect super modules that consist of several subcellular modules performing specific biological functions among cell compartments [13]. Super modules are more functionally diverse and have been verified to be more associated with known protein complexes and biological pathways in multiple PPI resources. Locational and topological overlap model (LTOM) requires the topological overlaps of a pair of proteins to be annotated in the same subcellular localization [14]. The module identified has good correspondence with the reference protein complexes and shows more relevance to cancers based on both human and yeast datasets.

**Table 1:** Dataset characteristics

Dataset	Tissue	Sepsis number	Control number	Platform
GSE95233	Whole blood	51	22	Affymetrix Human Genome U133 Plus 2.0 Array
GSE57065	Whole blood	28	25	Affymetrix Human Genome U133 Plus 2.0 Array
GSE28750	Whole blood	10	20	Affymetrix Human Genome U133 Plus 2.0 Array
GSE13904	Whole blood	52	18	Affymetrix Human Genome U133 Plus 2.0 Array
GSE9692	Peripheral blood	30	15	Affymetrix Human Genome U133 Plus 2.0 Array
GSE8121	Whole blood	15	60	Affymetrix Human Genome U133 Plus 2.0 Array

On top of this methods, weighted gene co-expression network analysis (WGCNA) is a widely used module identification method especially for studying biological networks based on pairwise correlations between transcriptome discoveries [26]. It classifies the transcriptome into biologically meaningful modules of co-expressed genes linked to specific cell types, organelles, and biological pathways. Co-expression modules also link to disease processes in which the most centrally connected genes are highly enriched for key drivers that play prominent roles in disease pathogenesis.

**Fig. 1:**
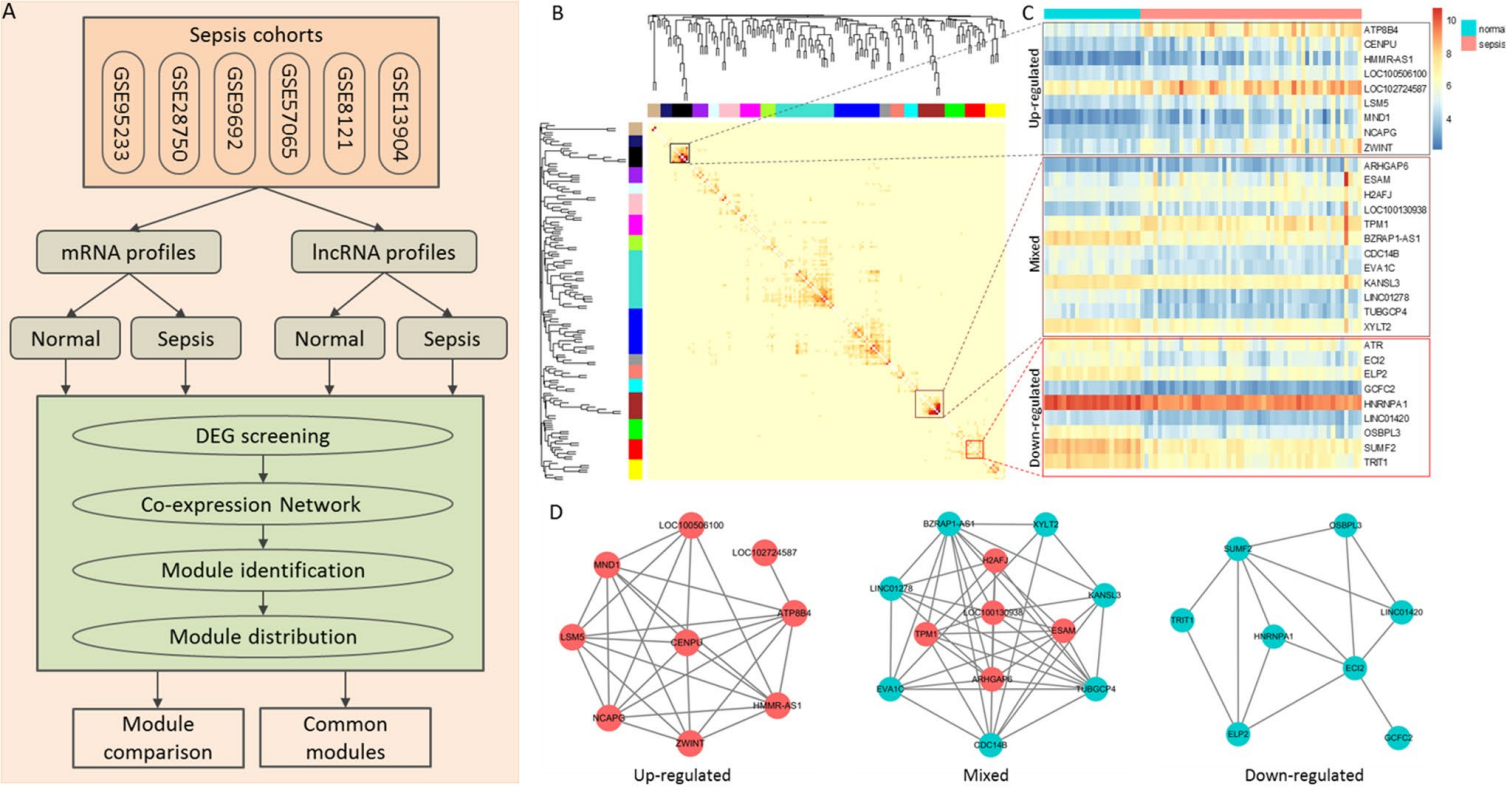
Module identification and definition. (A) Flowchart of the study. Both mRNAs and lncRNAs are investigated. (B) Identification of co-expression modules from the topological overlap matrix of GSE95233 using WGCNA. Modules are colored by the side bars. (C) Expression pattern of the genes in three representative modules, an up-regulated, a down-regulated, and a mixed one. Cyan and pink bars on top of the heatmap represent normal and sepsis samples, respectively. (D) Three sub-networks representing up-regulated, mixed, and down-regulated module, respectively. Modules containing over 90% up-regulated genes were defined as up-regulated modules while modules including more than 90% down-regulated genes were down-regulated modules

In this study, we aim to investigate the expression homogeneity of co-expression modules for both coding and non-coding genes in sepsis. We constructed gene co-expression networks and identified gene modules using WGCNA based on differentially expressed findings from six sepsis datasets. Subsequently, we characterized the co-expression pattern of lncRNAs and mRNAs and compared the homogeneity of the co-expression modules between sepsis and normal state. Finally, we selected modules that shared the highest number of genes across datasets as consistent modules associated with sepsis, and we identified common genes within these modules for further functional analysis and discussion.

## Materials and methods

### Preprocessing of raw data

We collected three adult microarray expression datasets, GSE28750, GSE57065, and GSE95233, and three children datasets, GSE8121, GSE9692, and GSE13904, from the Gene Expression Omnibus (GEO) database [27]. All these datasets were based on the Affymetrix GPL570 platform (Affymetrix Human Genome U133 Plus 2.0 Array). The characteristics of these datasets is provided in Table 1. The raw data for each dataset was normalized by means of the Robust Multi-Array Average (RMA) using the “affy” package of Bioconductor platform in in R environment (version 3.61) [28, 29]. Replicated genes were averaged and genes with multiple symbols were filtered out [30, 31], resulting in 21,655 genes for subsequent analysis.

### Reannotation of microarray platform

To explore how the lncRNAs are expressed in sepsis, we reannotated lncRNAs based on the six microarray datasets, which were originally built for quantifying the expression intensity of mRNAs. The Affymetrix GPL570 platform has been widely used for gene expression profiling of a variety of diseases and it has the most comprehensive coverage of the annotated human lncRNAs. Using the latest NetAffx Annotation File, HG-U133_Plus_2 Annotations (Release 35, 04/16/15) [32], we reannotated the lncRNAs of these datasets as follows [33–35]: (1) The Refseq ID labeled with NR_ or XR_, indicative of non-coding RNAs, are retained; (2) the Ensemble gene IDs annotated with antisense, processed transcripts, sense overlapping, non-sense mediated decay, sense intronic or lincRNA are retained; and (3) pseudogenes, rRNAs, microRNAs, and other small RNAs including tRNAs, snRNAs and snoRNAs are filtered out. Finally, 5,016 probesets were detected as lncRNAs representing 3,640 unique lncRNAs. For the replicated lncRNAs, we summarized them using the average expression values.

**Table 2:** Summary of differentially expressed mRNAs and lncRNAs.

Datasets	Differentially expressed genes(%)	Up-regulated DEGs(%)	Down-regulated DEGs(%)	Differentially expressed lncRNAs(%)	Up-regulated DELs(%)	Down-regulated DELs(%)
GSE28750	2382(10.99)	1155(48.49)	1227(51.51)	280(8.70)	104(37.14)	176(62.86)
GSE57065	2578(11.90)	1156(44.84)	1422(55.16)	316(9.81)	122(38.61)	194(61.39)
GSE95233	3497(16.15)	1888(53.99)	1609(46.01)	431(13.39)	206(47.80)	225(52.20)
GSE8121	2085(9.63)	846(40.58)	1239(59.42)	245(7.60)	75(30.61)	170(69.39)
GSE9692	2507(11.58)	1148(45.79)	1359(54.21)	277(8.60)	110(39.71)	167(60.29)
GSE13904	1385(6.40)	771(55.67)	614(44.33)	140(4.35)	61(43.57)	79(56.43)

The table represents the numbers (and percentages in parenthesis) of DEGs and DELs between patients with sepsis and healthy people. The direction of regulation (up or down-regulation) of the genes was also specified

### Co-expression network construction and module detection

A gene or a lncRNA is considered as significantly differentially expressed if the two tailed t-test p value was less than 0.05 and the absolute fold change was larger than 1.5. Weighted correlation network analysis (WGCNA) was used for co-expression network construction and module detection [14, 26]. We first calculated the Pearson Correlation Coefficients (PCC) between any possible pair of genes to generate a co-expression network. Then, a power function $f\left(x\right)={x}^{b}$ was applied to adjust the co-expression network to be scale-free. A common linear model that regressed the network degree is used to evaluate whether the degree distribution follows a power law. After that, the weighted co-expression network (or adjacent matrix) is transformed into a topological overlap matrix (TOM), which is a classical algorithm considering both direct and indirect interactions of all the vertexes (mRNAs or lncRNAs) in the network, resulting in biologically more meaningful modules. The co-expressed modules were identified using hierarchical clustering tree with different colors, and the module structure was displayed by both topological overlapping matrix and co-expression network.

We built the co-expression networks of differentially expressed mRNAs (DEGs) and lncRNAs (DELs) for sepsis samples and healthy samples, respectively. The minimum module size is set as ten for mRNA data and five for lncRNA data, due to lncRNA is much less than mRNAs. A module is defined as up-regulated (or down-regulated) if more than 95% of the module members are up-regulated (or down-regulated) (Fig. 1C, D). The gene pairs with the absolute PCC > 0.7 for DEGs and (absolute PCC > 0.5 for DELs) were considered to be strongly co-expressed. The co-expression module networks were visualized by Cytoscape (version 3.1.0) [36].

**Table 3:** Summary of mRNA coexpression modules

Datasets	Sepsis modules	Up-regulated modules(%)	Mixed gene modules(%)	Down-regulated modules(%)	Normal modules	Up-regulated modules(%)	Mixed gene modules(%)	Down-regulated modules(%)
GSE28750	57	3(5.26)	48(84.21)	6(10.52)	34	4(11.76)	29(85.3)	1(2.94)
GSE57065	54	15(27.78)	21(38.89)	18(33.33)	49	3(6.12)	42(85.7)	4(8.16)
GSE95233	55	26(47.27)	13(23.64)	16(29.09)	60	9(15.00)	42(70.0)	9(15.00)
GSE8121	32	13(40.62)	7(21.88)	12(37.50)	43	9(20.93)	20(46.5)	14(32.56)
GSE9692	55	17(30.91)	12(21.82)	26(47.27)	41	3(7.31)	34(82.9)	4(9.76)
GSE13904	29	17(58.62)	2(6.90)	10(34.48)	33	16(48.48)	13(39.4)	4(12.12)

### Identification of common modules

To select genes for further analyses, modules sharing common genes in different datasets were identified. These genes are consistently involved in the co-expression modules and working together to perform specific biological functions, which might play important roles in the pathogenesis and prognosis of sepsis. The main procedure to detect the common module among multiple datasets consists of the three steps: (1) identify the overlapping genes among all datasets; (2) calculate the percentage of overlapping genes in each module, i.e., the number of overlapping genes over the module size; and (3) identify common modules with a high overlapping percentage. Finally, we obtained three common co-expression modules among five datasets except GSE28750, one up-regulated module with 8 overlapping DEGs, one down-regulated module with 11 overlapping DEGs, and one down-regulated module with 2 overlapping DELs.

**Table 4:** Summary of lncRNA coexpression modules

Datasets	Sepsis modules	Up-regulated modules(%)	Mixed gene modules(%)	Down-regulated modules(%)	Normal modules	Up-regulated modules(%)	Mixed gene modules(%)	Down-regulated modules(%)
GSE28750	15	1(6.67)	10(66.67)	4(26.67)	13	1(7.69)	9(69.23)	3(23.08)
GSE57065	17	6(35.29)	8(47.06)	3(17.65)	13	0(0)	12(92.31)	1(7.69)
GSE95233	17	8(47.06)	2(11.76)	7(41.18)	24	6(25.00)	13(54.17)	5(20.83)
GSE8121	12	3(25.00)	1(8.33)	8(66.67)	14	3(21.43)	6(42.86)	5(35.71)
GSE9692	13	3(23.08)	5(38.46)	5(38.46)	13	1(7.69)	11(84.62)	1(7.69)
GSE13904	5	1(20.00)	4(80.00)	0(0)	6	2(33.33)	2(33.33)	2(33.33)

### Function enrichment analysis

Gene Ontology (GO) is the most widely used biological ontology that consists of three domains, biological processes, cellular components, and molecular functions [37]. GO enrichment analysis was usually carried out to facilitate elucidating the biological implications of a set of interesting coding genes, such as differentially expressed genes [38]. We used an R package *clusterProfiler* to perform the enrichment analysis to achieve related biological processes for a given set of genes [39]. The number of genes detected by the platform (GPL570, n = 21,655) was used as the background gene list.

Thus far, no ontology has been developed for direct enrichment analysis of lncRNAs, owing to the incompleteness of lncRNA annotation. In this study, we annotated lncRNAs according to the functions of their co-expressed mRNAs. Specifically, Pearson Correlation Coefficients (PCCs) were calculated between a lncRNA and all the mRNAs, and then the top 15 mRNAs with the highest absolute PCCs were selected to represent the lncRNA for functional enrichment.

## Results

### Differential analysis of coding and non-coding genes

We analyzed six gene expression datasets of whole blood and peripheral blood mononuclear cell (PBMC) for patients with sepsis (Fig. 1A). All of these datasets included control blood samples of the healthy individuals. To identify probes with lncRNA annotation, the probes were mapped to the latest NetAffx Annotation File (HG-U133_Plus_2 Annotations, Release 35) [32]. Some probes originally annotated as protein-coding genes were leveraged to represent antisense, processed transcripts, sense overlapping, non-sense mediated decay, sense intronic, or lincRNA. Finally, 5,016 probesets were detected representing 3,640 unique lncRNAs.

**Fig. 2:**
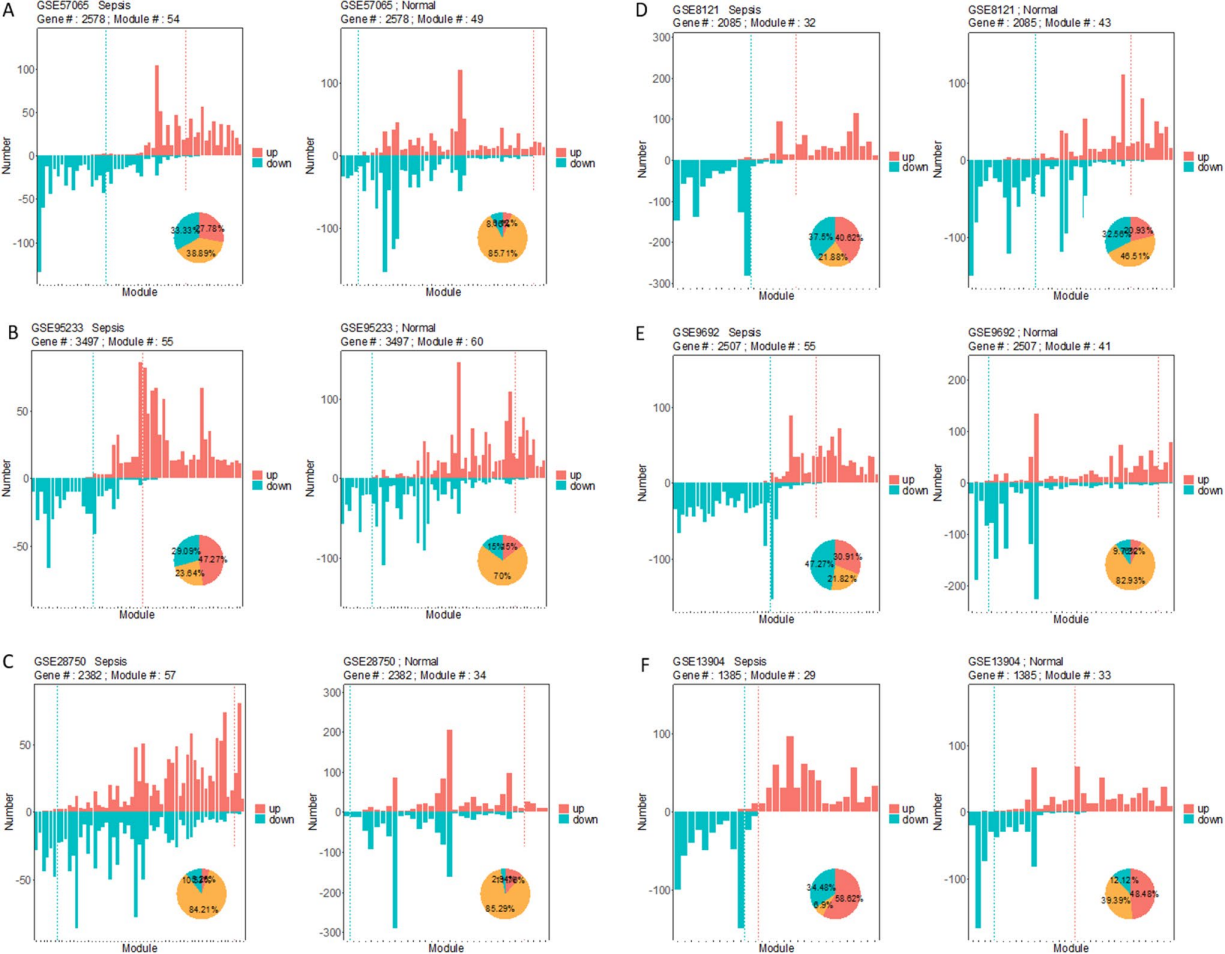
Composition of mRNA modules identified from different states. Y axis represents the number of up or down-regulated mRNAs in each module. Different module types are separated by the dashed lines. The embedded pie shows the proportions of each type of modules

By comparing the gene expression levels between sepsis samples and controls, differentially expressed genes (DEGs) and differentially expressed lncRNAs (DELs) were identified in each dataset. The differential analysis reported statistically significant alterations (P-value < 0.05 and fold-change > 1.5) in 6.4-16.15% of mRNAs (11.12% in average) and 4.35–13.39% of lncRNAs (8.74% in average). Specifically, 2382, 2578, 3497, 2085, 2507, and 1385 DEGs were screened from GSE28750, GSE57065, GSE95233, GSE8121, GSE9692, and GSE13904, respectively (Table 2). 412 up-regulated and 300 down-regulated genes out of them were consistently detected as DEG in all the six datasets (Supplementary Fig. 1). Additionally, we identified 280, 316, 431, 245, 277, and 140 DELs from the six datasets, respectively, and 70 (31 up-regulated and 39 down-regulated) out of them were commonly detected by all these datasets. The ratio of differentially expressed discoveries for lncRNAs is slightly lower than that of mRNAs (1.92% vs. 3.29%).

**Fig. 3:**
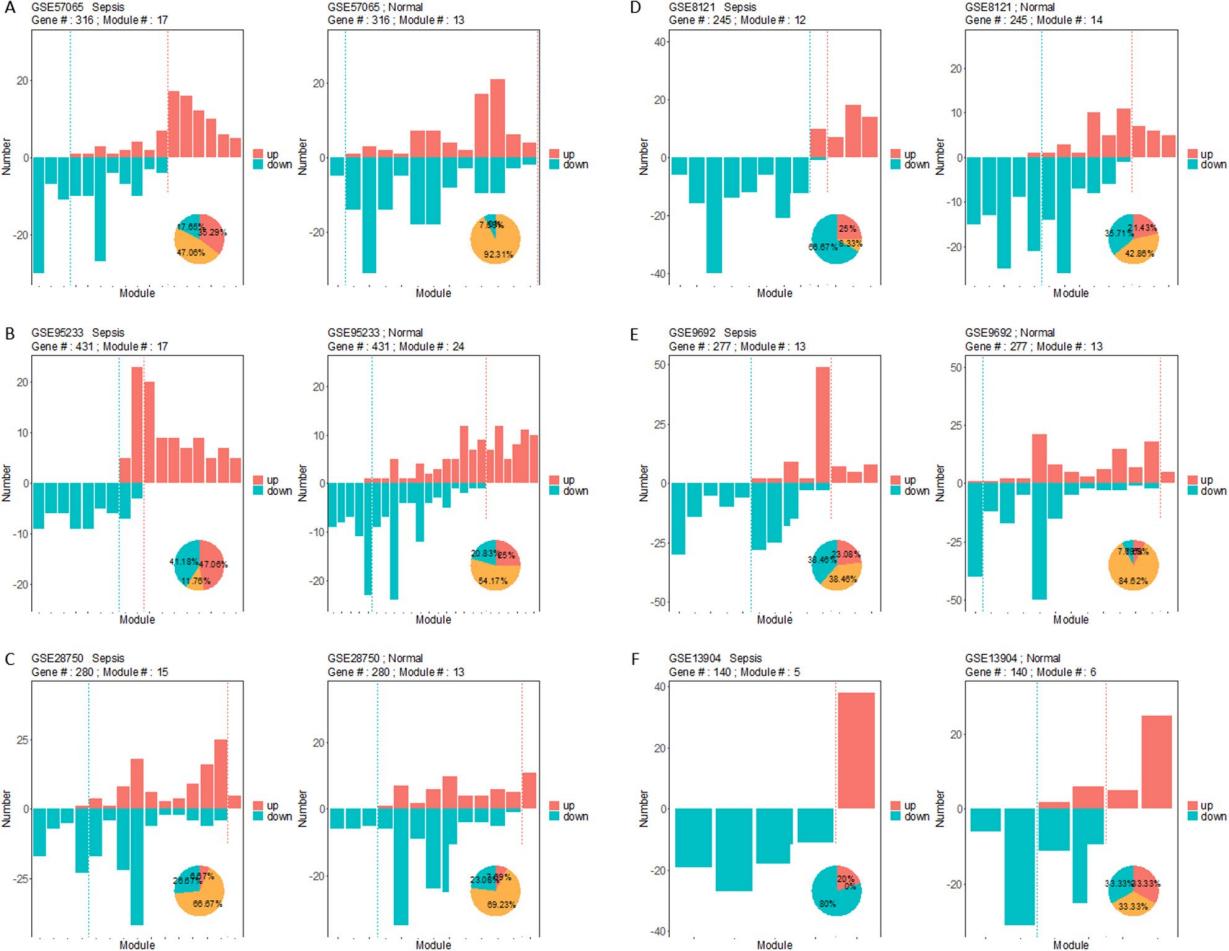
Composition of lncRNA modules identified from different states. Y axis represents the number of up or down-regulated lncRNAs in each module. Different module types are separated by the dashed lines. The embedded pie shows the proportions of each type of modules

### Homogeneity of mRNA modules in sepsis

For each gene expression dataset, sepsis co-expression networks and normal co-expression networks were separately conducted based on the differentially expressed mRNAs. Modules were identified from these co-expression networks using WGCNA [26] (Fig. 1B). Each color represented a type of module and we extracted the gene in each module (Tables 3 and 4). In the sepsis state, 57, 54, 55, 32, 55 and 29 gene modules were identified from GSE28750, while the numbers were 34, 49, 60, 43, 41, and 33 in the normal state. The modules were stratified into three groups, up-regulated, down-regulated and mixed modules, based on the proportion of up- and down-regulated genes (Fig. 1D). Modules containing over 90% up-regulated genes were defined as up-regulated modules while modules including more than 90% down-regulated genes were down-regulated modules.

**Fig. 4:**
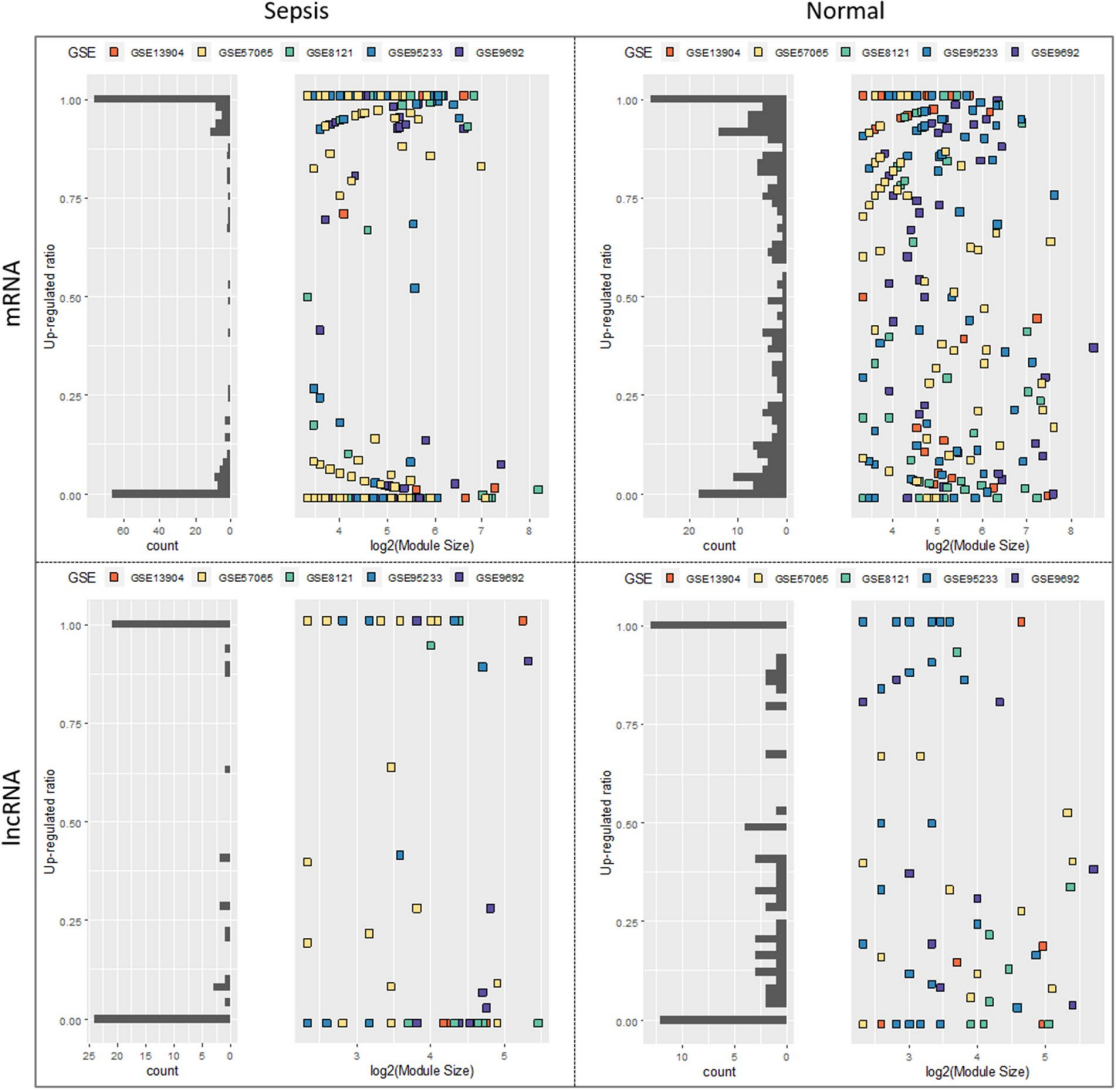
Comparison of the expression patterns of mRNA and lncRNA modules in the sepsis and healthy state. In each panel, bar plot represents the distribution of the modules in terms of the up-regulated ratio of the module members, while the point plot corresponds to the modules distributed on two dimensions, up-regulated ratio and log2 transferred module size. Horizontal axis shows log2 (module size) and vertical axis represents the up regulated ratio of module

**Fig. 5:**
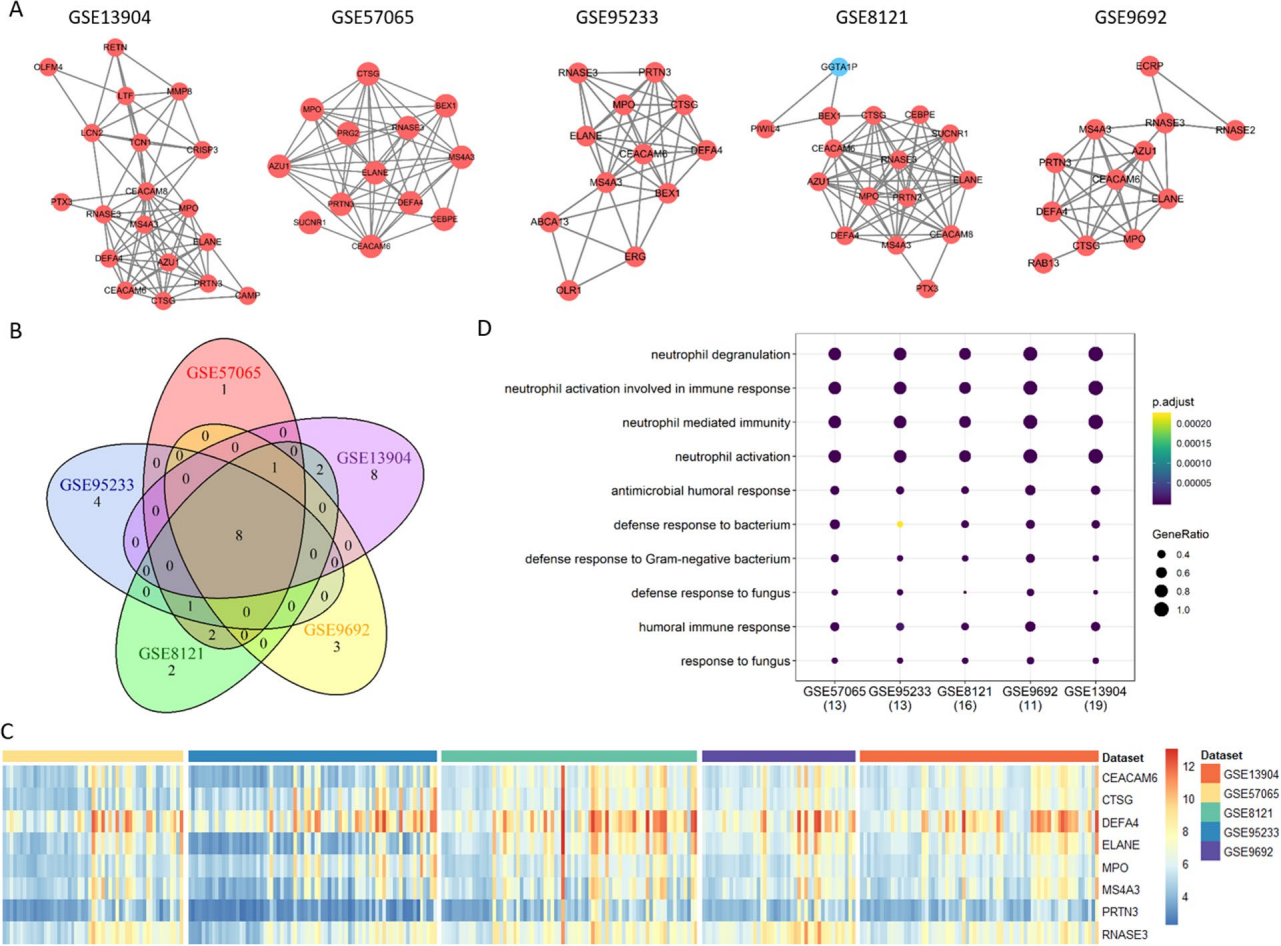
Up-regulated mRNA modules. (A) Common up-regulated modules identified from five different datasets. (B) Venn diagram of genes from the five common up-regulated modules. (C) Expression heatmap of eight common up-regulated mRNAs in the five datasets. (D) Functional analysis of the eight common up-regulated mRNAs.

In Fig. 2, the bar chart shows the number of up-regulated DEGs (red) and down-regulated DEGs (cyan) in each module, while the pie graph represents the percentage of the up-regulated module (red), down-regulated module (cyan), and mixed module (yellow) in each dataset. We observed that the sepsis gene modules tend to be more homogeneous than the normal ones. Namely, a majority of sepsis gene modules are either up-regulated or down-regulated and only a small fraction of them with mixed expression direction, whereas the normal modules consist of more mixed modules and the proportion of up- and down-regulated modules are relatively low (Fig. 2). Specifically, 15 (27.78%) up-regulated, 18 (33.33%) down-regulated, and 21(38.89%) mixed modules were detected in the sepsis state for dataset GSE57065, while the figures are 3 (6.12%), 4 (8.16%), and 42 (85.7%) in the normal state. Similar findings were produced for all the other datasets except GSE28750.

**Fig. 6:**
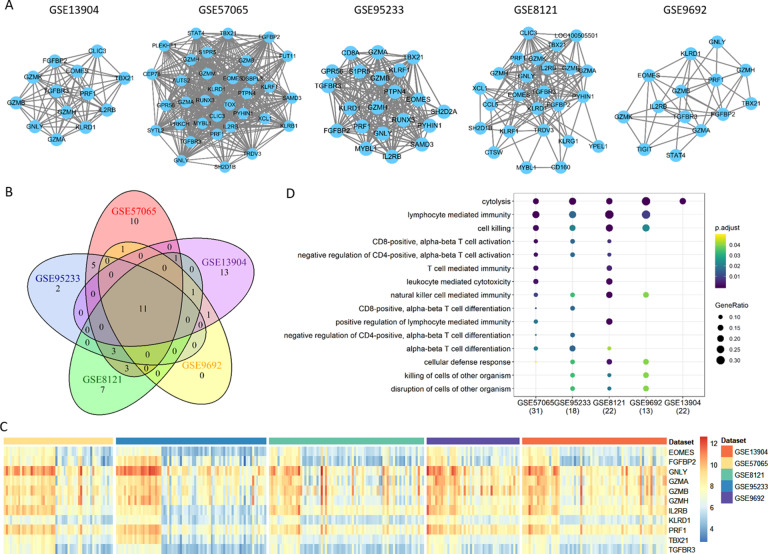
Down-regulated mRNA modules. (A) Common down-regulated modules identified from five different datasets. (B) Venn diagram of genes from the five common down-regulated modules. (C) Expression heatmap of the 11 common down-regulated mRNAs in the five datasets. (D) Functional analysis of the 11 common down-regulated mRNAs.

**Fig. 7:**
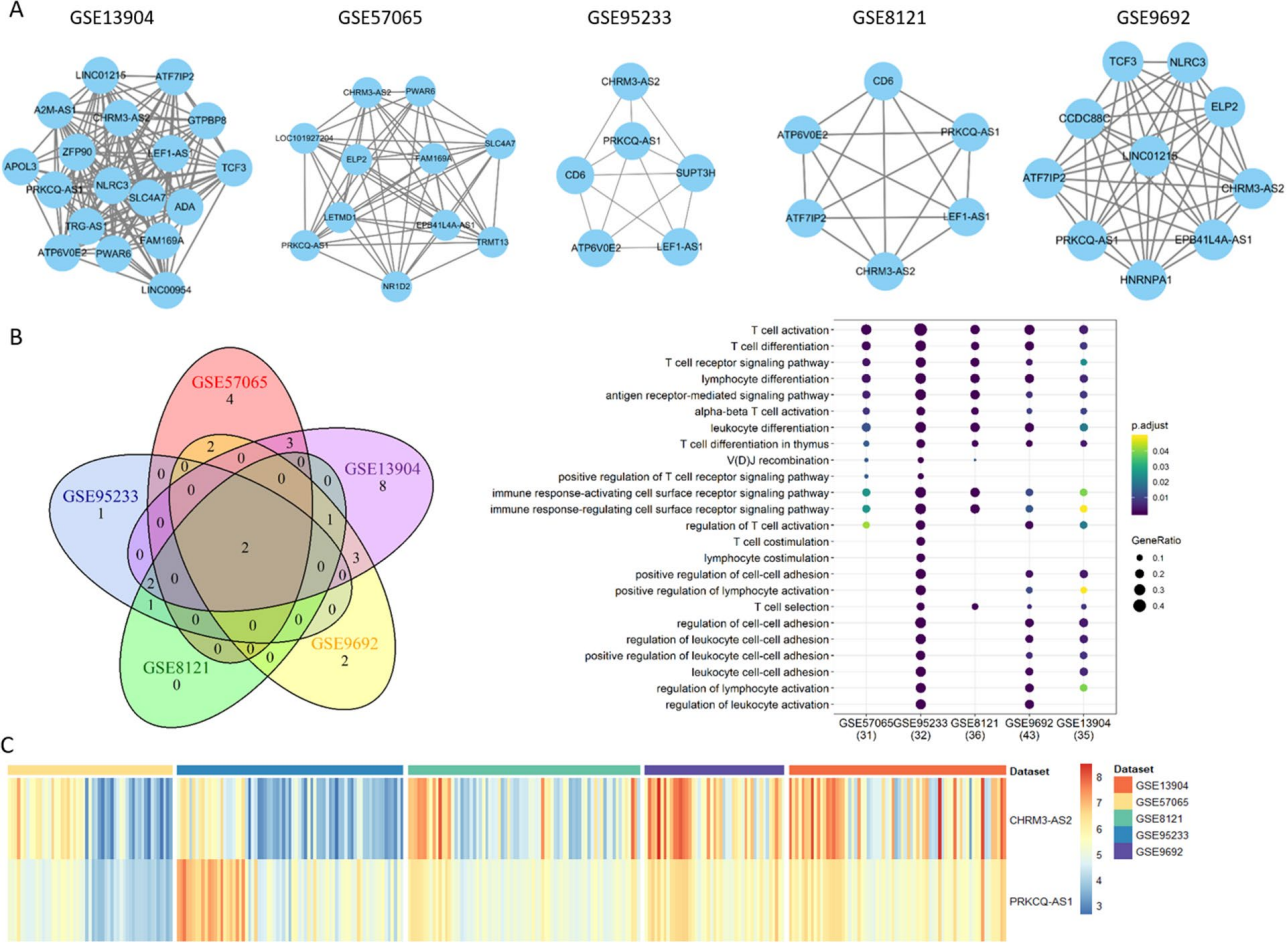
Down-regulated lncRNA modules. (A) Common down-regulated modules identified from five different datasets. (B) Venn diagram of lncRNAs from the five common down-regulated modules. (C) Expression heatmap of the two common down-regulated lncRNAs in the five datasets. (D) Functional analysis of the two common down-regulated lncRNAs. Co-expressed mRNAs of each lncRNA are used for function enrichment

### Homogeneity of lncRNA modules in sepsis

We draw the same conclusions from the lncRNA modules. The sepsis lncRNA modules were more homogeneous in comparison to the normal ones (Fig. 3). Specifically, 57, 54, 55, 32, 55, and 29 gene modules were identified from GSE28750 of sepsis state, while in the normal state the numbers were 34, 49, 60, 43, 41, and 33, respectively (Table 4). In GSE57065, for instance, 15 (27.78%) up-regulated, 18 (33.33%) down-regulated, and 21(38.89%) mixed modules were screened in the sepsis state, while the numbers were 3 (6.12%), 4 (8.16%), and 42 (85.7%) in normal condition. Similar findings were made for all these datasets except GSE28750.

**Table 5:** Discription of the functions of down-regulated genes

Gene	Function discription
EOMES	Proteins encoded by EOMES may be necessary for the differentiation of effector CD8 + T cells which are involved in defense against viral infections.
FGFBP2	The encoded protein is a serum protein that is selectively secreted by cytotoxic lymphocytes and may be involved in cytotoxic lymphocyte-mediated immunity.
GNLY	The product of GNLY is a member of the saposin-like protein (SAPLIP) family and is located in the cytotoxic granules of T cells, which are released upon antigen stimulation.
GZMA	The encoded protein is a T cell- and natural killer cell-specific serine protease that may function as a common component necessary for lysis of target cells by cytotoxic T lymphocytes and natural killer cells.
GZMB	The encoded preproprotein is secreted by natural killer (NK) cells and cytotoxic T lymphocytes (CTLs) and proteolytically processed to generate the active protease, which induces target cell apoptosis. This protein also processes cytokines and degrades extracellular matrix proteins, and these roles are implicated in chronic inflammation and wound healing.
GZMH	It is reported to be constitutively expressed in the NK (natural killer) cells of the immune system and may play a role in the cytotoxic arm of the innate immune response by inducing target cell death and by directly cleaving substrates in pathogen-infected cells.
IL2RB	The interleukin 2 receptor (IL2RB) is involved in T cell-mediated immune responses and it is primarily expressed in the hematopoietic system.
KLRD1	KLRD1 (CD94) is an antigen preferentially expressed on Natural killer (NK) cells, which are a distinct lineage of lymphocytes that mediate cytotoxic activity and secrete cytokines upon immune stimulation.
PRF1	Protein PRF1 is structurally similar to complement component C9 that is important in immunity. This protein forms membrane pores that allow the release of granzymes and subsequent cytolysis of target cells. Mutations in this gene are associated with a variety of human diseases.
TBX21	TBX21 is the human ortholog of mouse Tbx21/Tbet gene. Studies in mouse show that Tbx21 protein is a Th1 cell-specific transcription factor that controls the expression of the hallmark Th1 cytokine, interferon-gamma (IFNG). Expression of the human ortholog also correlates with IFNG expression in Th1 and natural killer cells, suggesting a role for this gene in initiating Th1 lineage development from naive Th precursor cells.
TGFBR3	TGFBR3 encoded receptor is a membrane proteoglycan that often functions as a co-receptor with other TGF-beta receptor superfamily members.

To provide an overview of the distributions of different types of mRNA modules and lncRNA modules, we calculated the up-regulated gene ratio of each module and sought to compare the ratio between different states (Fig. 4). Dataset GSE28750 was excluded due to its expression pattern was different from that of the other datasets. In Fig. 4, a square represents a module and color represents dataset. The vertical axis represents the up-regulated ratio while the horizontal axis shows the number of modules. Interestingly, the lncRNAs in most of the sepsis modules are exclusively up-regulated or down-regulated with an extremely high homogeneity, whereas the lncRNA modules are more heterogeneous in the normal state. In addition, the distributions of module number are consistent regardless of the module size in either the sepsis or the normal state, indicating the expression homogeneity is independent of the module size (Fig. 4).

### Identification of consistent coding and non-coding genes

Generally, the identified differentially expressed molecules and co-expression modules are inconsistent across different datasets. To address this issue, we screened the sepsis modules to obtain the ones with the maximum number of common genes across the five datasets (Fig. 5A). Five up-regulated consistent modules were identified with eight common genes, i.e., CEACAM6, CTSG, DEFA4, ELANE, MPO, MS4A3, PRTN3, and RNASE3 (Fig. 5B **and C**). All of those genes are involved in biological processes of neutrophil degranulation, neutrophil activation involved in immune response, neutrophil mediated immunity, neutrophil activation, etc., practically all of which are neutrophil related immune functions (Fig. 5D). For the identified down-regulated consistent modules, 11 common genes are shared, including EOMES, FGFBP2, GNLY, GZMA, GZMB, GZMH, IL2RB, KLRD1, PRF1, TBX21, and TGFBR3 (Fig. 6). Interestingly, those genes are mainly implicated in T cell related immune functions, such as lymphocyte mediated immunity, cell killing, T cell activation, and T cell mediate immunity.

Similarly, for the lncRNA modules, the consistent modules detected from the five datasets share two lncRNAs, CHRM3 antisense RNA 2 (CHRM3-AS2) and PRKCQ Antisense RNA 1 (PRKCQ-AS1). Their module members are densely connected and consistently down-regulated (Fig. 7). In analogy to the down-regulated genes, the genes co-expressed with these lncRNAs mainly represent in T cell related immune functions, including T cell activation, T cell deferrization, T cell reporter signaling pathway, lymphocyte differentiation, etc. Our findings indicate that the up-regulated genes are more likely to function in neutrophil related immune functions, while the genes in the down-regulated modules tend to participate in T cell related immune functions, either coding or non-coding genes.

## Discussions

We initially identified genes and lncRNAs that exhibited significant differential expression between sepsis and normal states in six transcriptome datasets. Using these differentially expressed findings, we constructed co-expression networks and identified gene co-expression modules. Our analysis revealed that sepsis modules displayed a more homogeneous expression pattern, predominantly consisting of either up-regulated or down-regulated genes, while a substantial portion of normal modules exhibited a mixed pattern, with up- and down-regulated genes evenly distributed. Among these modules, we identified eight up-regulated and 11 down-regulated common genes that were consistently observed across diverse datasets, indicating shared information. Remarkably, all these genes were involved in human immunological pathways. The up-regulated genes mainly function in neutrophil whereas the down-regulated ones usually regulate T cell. Also, two down-regulated lncRNAs CHRM3-AS2 and PRKCQ-AS1, were determined as sepsis associated lncRNAs functioning in T cell activation and differentiation.

In sepsis, for either coding or non-coding modules, a majority of genes have the same expression direction, revealing that genes in a module are under- or over-expressed together to function in some specific biological processes like immunity and inflammation. Our results show that ten out of the 11 genes consistently under-expressed in sepsis modules may function in T cell mediated pathways (Table 5). For instance, proteins encoded by EOMES may be necessary for the differentiation of effector CD8 + T cells which are involved in defense against viral infections. The one left is TGFBR3, the receptor encoded by which is a membrane proteoglycan that often functions as a co-receptor with other TGFβ receptor superfamily members [40]. TGFβ has a wide range of activity regulating various immune cells with soluble TGFBR3 potentially inhibiting TGFβ signaling [41, 42].

lncRNAs can bind to DNA, RNA and proteins depending on sequence and chromatin structure, thereby affecting RNA splicing, stability and translation, and ultimately modulating the expression of target genes in numerous pathophysiological processes such as disorders of immune system [20, 43], but their role in sepsis-induced immunity has not been explored. Owing to microarray platforms include probes representing lncRNAs, we reannotated lncRNAs and established lncRNA expression profilings. Through constructing and analyzing co-expression modules at different states using the screened differentially expressed lncRNAs, we found two novel lncRNAs are associated with sepsis, CHRM3-AS2 and PRKCQ-AS1. In analogy to the down-regulated genes, they are involved in sepsis pathogenesis pathways, such as T cell receptor signaling pathway, T cell, lymphocyte, and leukocyte differentiation, indicating the critical role of lncRNAs in sepsis initiation and progression. Our results provide evidence that lncRNAs have a significant impact on immune responses induced by inflammation in addition to mRNA (Fig. 6).

Specifically, the protein coding by interleukin 2 receptor (IL2RB), a member of the down-regulated modules, is interacted with PRKCQ-AS1, which has already been reported to be involved in T cell functions and play a key role in immunology [44, 45]. IL2RB is involved in T cell-mediated immune responses and it is primarily expressed in the hematopoietic system, which is tightly connected to the immune system [46]. The regulatory mechanism of lncRNA PRKCQ-AS1 on IL2RB need to be further explored to elucidate its function roles in sepsis. Proteins encoded by EOMES may be necessary for the differentiation of effector CD8 + T cells which are involved in defense against viral infections. lncRNA CHRM3-AS2 has been reported to be a potential regulator of EOMES [45]. The diagnosis and prognosis roles of the two lncRNAs and other module genes are also need to be systematically evaluated in our future work [47–51].

This study concentrated on co-expression pattern of mRNAs and lncRNAs in sepsis, providing a novel perspective and insight into sepsis coding and non-coding genes involved. This findings may facilitate the exploration of candidate therapeutic targets and molecular biomarkers for sepsis.

## Electronic supplementary material

Below is the link to the electronic supplementary material.

Supplementary Material 1

## Data Availability

Data are available at the GEO database (https://www.ncbi.nlm.nih.gov/geo/). Accession numbers are GSE28750, GSE57065, GSE95233, GSE8121, GSE9692, and GSE13904.

## References

[1] Rudd KE, Johnson SC, Agesa KM, Shackelford KA, Tsoi D, Kievlan DR, Colombara DV, Ikuta KS, Kissoon N, Finfer S (2020). Global, regional, and national sepsis incidence and mortality, 1990–2017: analysis for the global burden of Disease Study. Lancet.

[2] van der Poll T (2016). Future of sepsis therapies. Crit Care.

[3] Zheng X, Wu Q, Wu H, Leung KS, Wong MH, Liu X, Cheng L (2021). Evaluating the consistency of gene methylation in Liver Cancer using bisulfite sequencing data. Front Cell Dev Biol.

[4] Ho J, Chan H, Wong SH, Wang MH, Yu J, Xiao Z, Liu X, Choi G, Leung CC, Wong WT (2016). The involvement of regulatory non-coding RNAs in sepsis: a systematic review. Crit Care.

[5] Wang J, Zhang X, Cheng L, Luo Y (2020). An overview and metanalysis of machine and deep learning-based CRISPR gRNA design tools. RNA Biol.

[6] Wang J, Xiang X, Bolund L, Zhang X, Cheng L, Luo Y. GNL-Scorer: a generalized model for predicting CRISPR on-target activity by machine learning and featurization. J Mol Cell Biol 2020. 10.1093/jmcb/mjz116 PMC7883820 31900489

[7] Li L, Liu M, Yue L, Wang R, Zhang N, Liang Y, Zhang L, Cheng L, Xia J, Wang R (2020). Host-guest protein assembly for Affinity purification of Methyllysine Proteomes. Anal Chem.

[8] Liu S, Zhao W, Liu X, Cheng L (2020). Metagenomic analysis of the gut microbiome in atherosclerosis patients identify cross-cohort microbial signatures and potential therapeutic target. FASEB J.

[9] Liu X, Zheng X, Wang J, Zhang N, Leung K-S, Ye X, Cheng L (2020). A long non-coding RNA signature for diagnostic prediction of sepsis upon ICU admission. Clin translational Med.

[10] Yang Y, Zhang Y, Li S, Zheng X, Wong MH, Leung KS, Cheng L. A robust and generalizable immune-related signature for sepsis diagnostics. IEEE/ACM Trans Comput Biol Bioinform 2021, PP. 10.1109/TCBB.2021.3107874 34437068

[11] Yin R, Liu X, Yu J, Ji Y, Liu J, Cheng L, Zhou J (2020). Up-regulation of autophagy by low concentration of salicylic acid delays methyl jasmonate-induced leaf senescence. Sci Rep.

[12] Cheng L, Liu P, Leung K-S. SMILE: A Novel Procedure for Subcellular Module Identification with Localization Expansion. In: *Proceedings of the 8th ACM International Conference on Bioinformatics, Computational Biology, and Health Informatics: 2017*: ACM; 2017: 754–755.

[13] Cheng L, Liu P, Leung KS (2018). SMILE: a novel procedure for subcellular module identification with localisation expansion. IET Syst Biol.

[14] Cheng L, Liu P, Wang D, Leung KS (2019). Exploiting locational and topological overlap model to identify modules in protein interaction networks. BMC Bioinformatics.

[15] Cheng L, Fan K, Huang Y, Wang D, Leung KS (2017). Full characterization of localization diversity in the human protein interactome. J Proteome Res.

[16] Wang R, Zheng X, Song F, Wong MH, Leung KS, Cheng L. Deciphering associations between gut microbiota and clinical factors using microbial modules. Bioinformatics 2023, 39(5). 10.1093/bioinformatics/btad213 PMC10191612 37084255

[17] Sweeney TE, Perumal TM, Henao R, Nichols M, Howrylak JA, Choi AM, Bermejo-Martin JF, Almansa R, Tamayo E, Davenport EE (2018). A community approach to mortality prediction in sepsis via gene expression analysis. Nat Commun.

[18] Scicluna BP, van Vught LA, Zwinderman AH, Wiewel MA, Davenport EE, Burnham KL, Nurnberg P, Schultz MJ, Horn J, Cremer OL (2017). Classification of patients with sepsis according to blood genomic endotype: a prospective cohort study. Lancet Respir Med.

[19] Zheng X, Leung KS, Wong MH, Cheng L (2021). Long non-coding RNA pairs to assist in diagnosing sepsis. BMC Genomics.

[20] Cheng L, Leung K-S (2018). Quantification of non-coding RNA target localization diversity and its application in cancers. J Mol Cell Biol.

[21] Liao Q, Xiao H, Bu D, Xie C, Miao R, Luo H, Zhao G, Yu K, Zhao H, Skogerbo G et al. ncFANs: a web server for functional annotation of long non-coding RNAs. Nucleic Acids Res 2011, 39(Web Server issue):W118–124. 10.1093/nar/gkr432 PMC3125796 21715382

[22] Ma L, Cao J, Liu L, Du Q, Li Z, Zou D, Bajic VB, Zhang Z (2019). LncBook: a curated knowledgebase of human long non-coding RNAs. Nucleic Acids Res.

[23] Liu X, Xu Y, Wang R, Liu S, Wang J, Luo Y, Leung KS, Cheng L (2021). A network-based algorithm for the identification of moonlighting noncoding RNAs and its application in sepsis. Brief Bioinform.

[24] Cheng L, Leung KS. Quantification of non-coding RNA target localization diversity and its application in cancers. J Mol Cell Biol. 2018;10(2):130–138. 10.1093/jmcb/mjy006 29390072

[25] Cheng L, Nan C, Kang L, Zhang N, Liu S, Chen H, Hong C, Chen Y, Liang Z, Liu X (2020). Whole blood transcriptomic investigation identifies long non-coding RNAs as regulators in sepsis. J Transl Med.

[26] Nepusz T, Yu H, Paccanaro A (2012). Detecting overlapping protein complexes in protein-protein interaction networks. Nat Methods.

[27] Langfelder P, Horvath S (2008). WGCNA: an R package for weighted correlation network analysis. BMC Bioinformatics.

[28] Edgar R, Domrachev M, Lash AE (2002). Gene expression Omnibus: NCBI gene expression and hybridization array data repository. Nucleic Acids Res.

[29] Irizarry RA, Hobbs B, Collin F, Beazer-Barclay YD, Antonellis KJ, Scherf U, Speed TP (2003). Exploration, normalization, and summaries of high density oligonucleotide array probe level data. Biostatistics.

[30] Liu X, Li N, Liu S, Wang J, Zhang N, Zheng X, Leung K-S, Cheng L. Normalization methods for the analysis of Unbalanced Transcriptome Data: a review. Front Bioeng Biotechnol 2019, 7(358). 10.3389/fbioe.2019.00358 PMC6988798 32039167

[31] Cheng L, Lo LY, Tang NL, Wang D, Leung KS (2016). CrossNorm: a novel normalization strategy for microarray data in cancers. Sci Rep.

[32] Cheng L, Wang X, Wong PK, Lee KY, Li L, Xu B, Wang D, Leung KS (2016). ICN: a normalization method for gene expression data considering the over-expression of informative genes. Mol Biosyst.

[33] Liu G, Loraine AE, Shigeta R, Cline M, Cheng J, Valmeekam V, Sun S, Kulp D, Siani-Rose MA (2003). NetAffx: Affymetrix probesets and annotations. Nucleic Acids Res.

[34] Zhou M, Zhao H, Wang X, Sun J, Su J (2019). Analysis of long noncoding RNAs highlights region-specific altered expression patterns and diagnostic roles in Alzheimer’s disease. Brief Bioinform.

[35] Zhou M, Hu L, Zhang Z, Wu N, Sun J, Su J (2018). Recurrence-Associated Long non-coding RNA signature for determining the risk of recurrence in patients with Colon cancer. Mol Ther Nucleic Acids.

[36] Peng F, Wang R, Zhang Y, Zhao Z, Zhou W, Chang Z, Liang H, Zhao W, Qi L, Guo Z (2017). Differential expression analysis at the individual level reveals a lncRNA prognostic signature for lung adenocarcinoma. Mol Cancer.

[37] Shannon P, Markiel A, Ozier O, Baliga NS, Wang JT, Ramage D, Amin N, Schwikowski B, Ideker T (2003). Cytoscape: a software environment for integrated models of biomolecular interaction networks. Genome Res.

[38] The Gene Ontology C (2019). The Gene Ontology Resource: 20 years and still GOing strong. Nucleic Acids Res.

[39] Cheng L, Leung K-S (2018). Identification and characterization of moonlighting long non-coding RNAs based on RNA and protein interactome. Bioinformatics.

[40] Yu G, Wang LG, Han Y, He QY (2012). clusterProfiler: an R package for comparing biological themes among gene clusters. OMICS.

[41] Nishida J, Miyazono K, Ehata S (2018). Decreased TGFBR3/betaglycan expression enhances the metastatic abilities of renal cell carcinoma cells through TGF-beta-dependent and -independent mechanisms. Oncogene.

[42] Lopez-Casillas F, Cheifetz S, Doody J, Andres JL, Lane WS, Massague J (1991). Structure and expression of the membrane proteoglycan betaglycan, a component of the TGF-beta receptor system. Cell.

[43] Kagamu H, Kitano S, Yamaguchi O, Yoshimura K, Horimoto K, Kitazawa M, Fukui K, Shiono A, Mouri A, Nishihara F (2020). CD4(+) T-cell immunity in the peripheral blood correlates with response to Anti-PD-1 therapy. Cancer Immunol Res.

[44] Liu X, Xu Y, Wang R, Liu S, Wang J, Luo Y, Leung KS, Cheng L. A network-based algorithm for the identification of moonlighting noncoding RNAs and its application in sepsis. Brief Bioinform 2020. 10.1093/bib/bbz154 32003790

[45] de Lima DS, Cardozo LE, Maracaja-Coutinho V, Suhrbier A, Mane K, Jeffries D, Silveira ELV, Amaral PP, Rappuoli R, de Silva TI (2019). Long noncoding RNAs are involved in multiple immunological pathways in response to vaccination. Proc Natl Acad Sci USA.

[46] Kang J, Tang Q, He J, Li L, Yang N, Yu S, Wang M, Zhang Y, Lin J, Cui T (2022). RNAInter v4.0: RNA interactome repository with redefined confidence scoring system and improved accessibility. Nucleic Acids Res.

[47] Danckwardt S, Tregouet DA, Castoldi E. Post-transcriptional control of hemostatic genes: mechanisms and emerging therapeutic concepts in thrombo-inflammatory disorders. Cardiovasc Res 2023. 10.1093/cvr/cvad046 PMC10325701 36943786

[48] Cheng L, Wu H, Zheng X, Zhang N, Zhao P, Wang R, Wu Q, Liu T, Yang X, Geng Q. GPGPS: a robust prognostic gene pair signature of glioma ensembling IDH mutation and 1p/19q co-deletion. Bioinformatics 2023, 39(1). 10.1093/bioinformatics/btac850 PMC9843586 36637205

[49] Wu Q, Zheng X, Leung KS, Wong MH, Tsui SK, Cheng L. meGPS: a multi-omics signature for hepatocellular carcinoma detection integrating methylome and transcriptome data. Bioinformatics 2022. 10.1093/bioinformatics/btac379 35674358

[50] Wang R, Zheng X, Wang J, Wan S, Song F, Wong MH, Leung KS, Cheng L. Improving bulk RNA-seq classification by transferring gene signature from single cells in acute myeloid leukemia. Brief Bioinform 2022. 10.1093/bib/bbac002 35136933

[51] Li H, Zheng X, Gao J, Leung KS, Wong MH, Yang S, Liu Y, Dong M, Bai H, Ye X (2022). Whole transcriptome analysis reveals non-coding RNA’s competing endogenous gene pairs as novel form of motifs in serous ovarian cancer. Comput Biol Med.

[52] Xu C, Li W, Li T, Yuan J, Pang X, Liu T, Liang B, Cheng L, Sun X, Dong S (2022). Iron metabolism-related genes reveal predictive value of acute coronary syndrome. Front Pharmacol.

