# Supplementary material for: Co-expression module analysis reveals high expression homogeneity for both coding and non-coding genes in sepsis
Source: BMC Genomics. 2023 Jul 24;24:418. doi: 10.1186/s12864-023-09460-9 (PMC10364430; doi:10.1186/s12864-023-09460-9)
Supplement: Supplementary file 1 — Supplementary Material 1 [file 12864_2023_9460_MOESM1_ESM.docx]

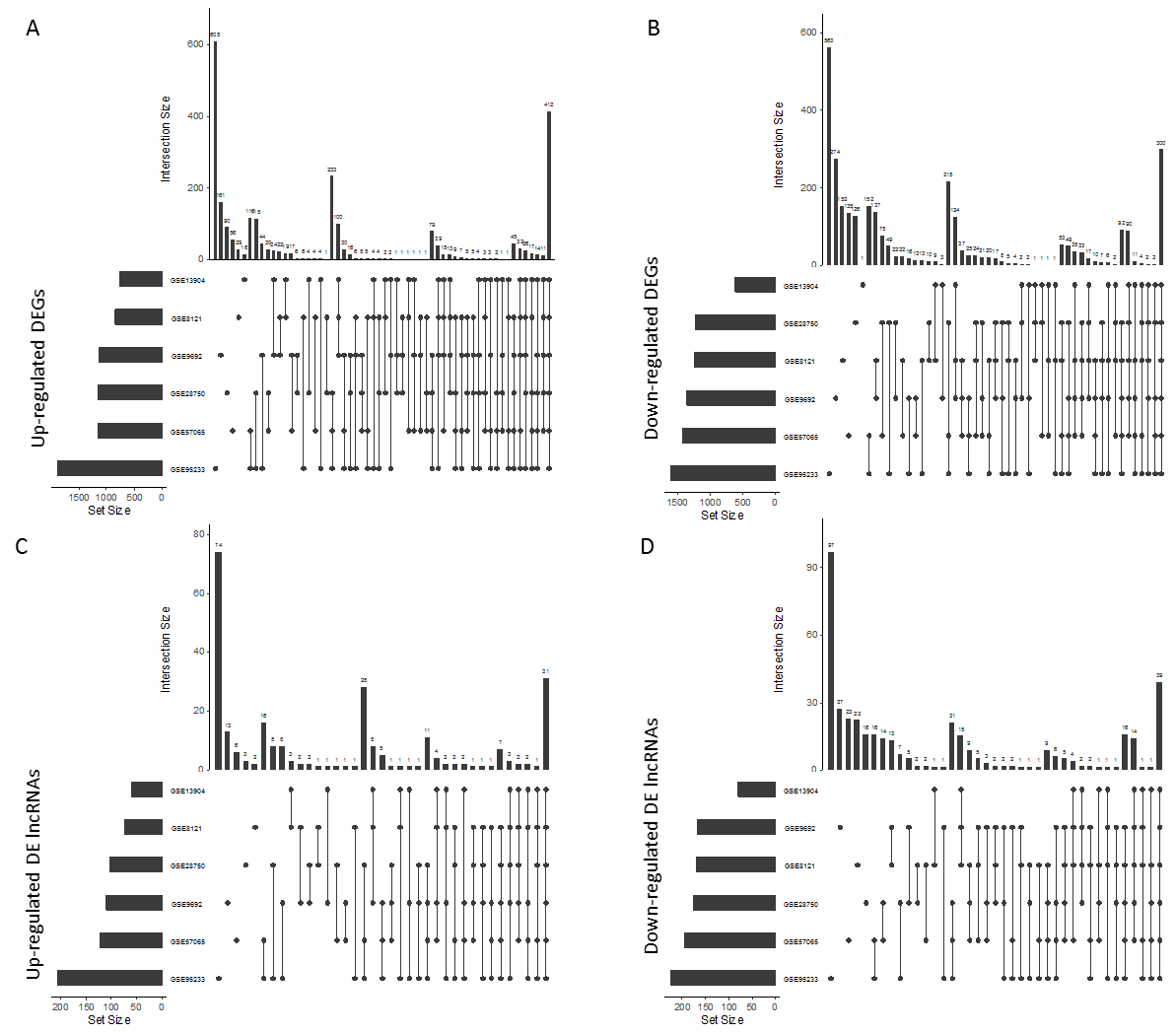


**Figure S1.** Overview of DEGs screened from different cohorts. The upset plots for the up-regulated DEGs (A), down-regulated DEGs (B), up-regulated DE lncRNAs (C), and down-regulated DE lncRNAs (D).

**
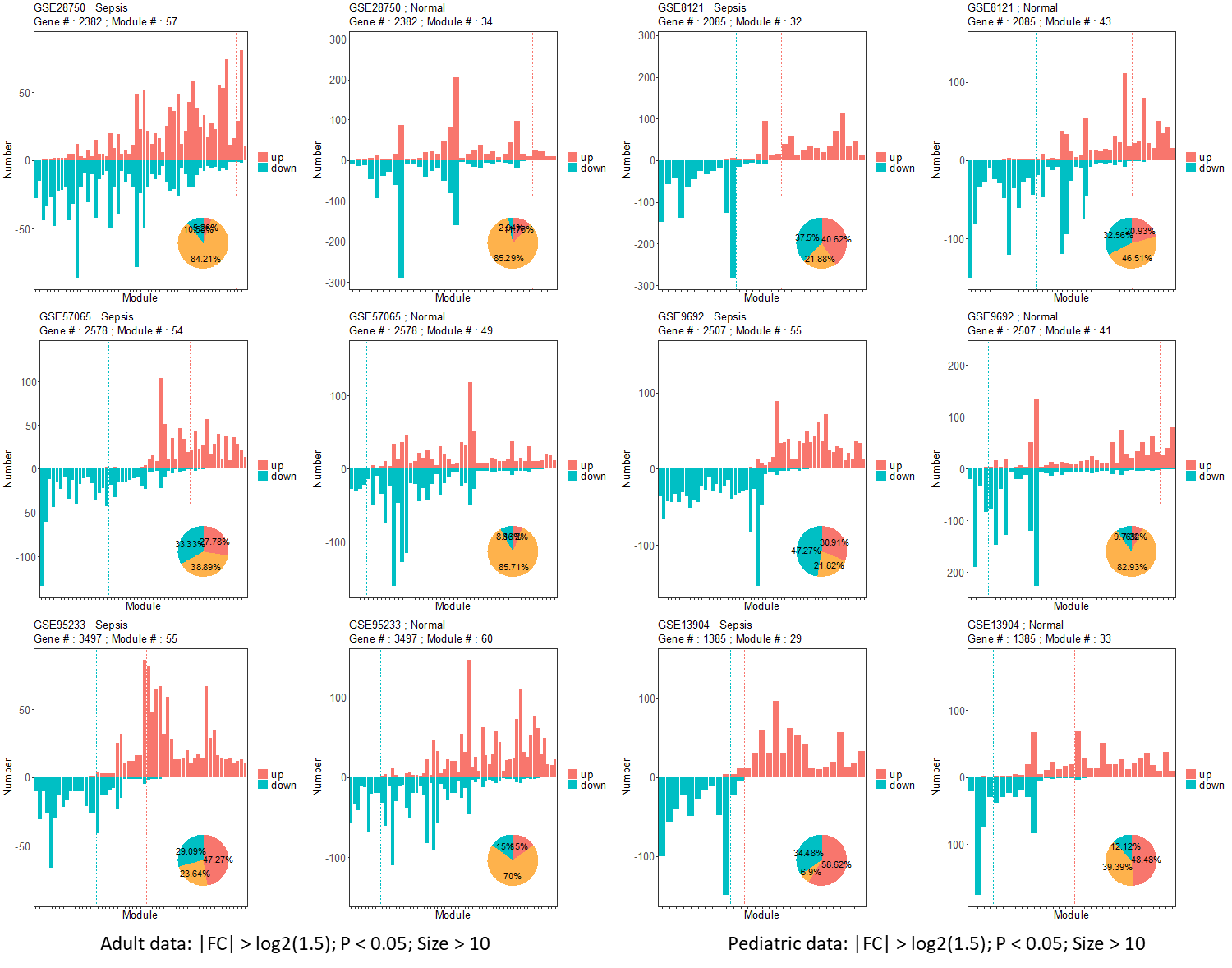
**

**Figure S2.** Composition of mRNA modules identified from different states. Y axis represents the number of up or down-regulated mRNAs in each module. Different module types are separated by the dashed lines. The embedded pie shows the proportions of each types of modules

**
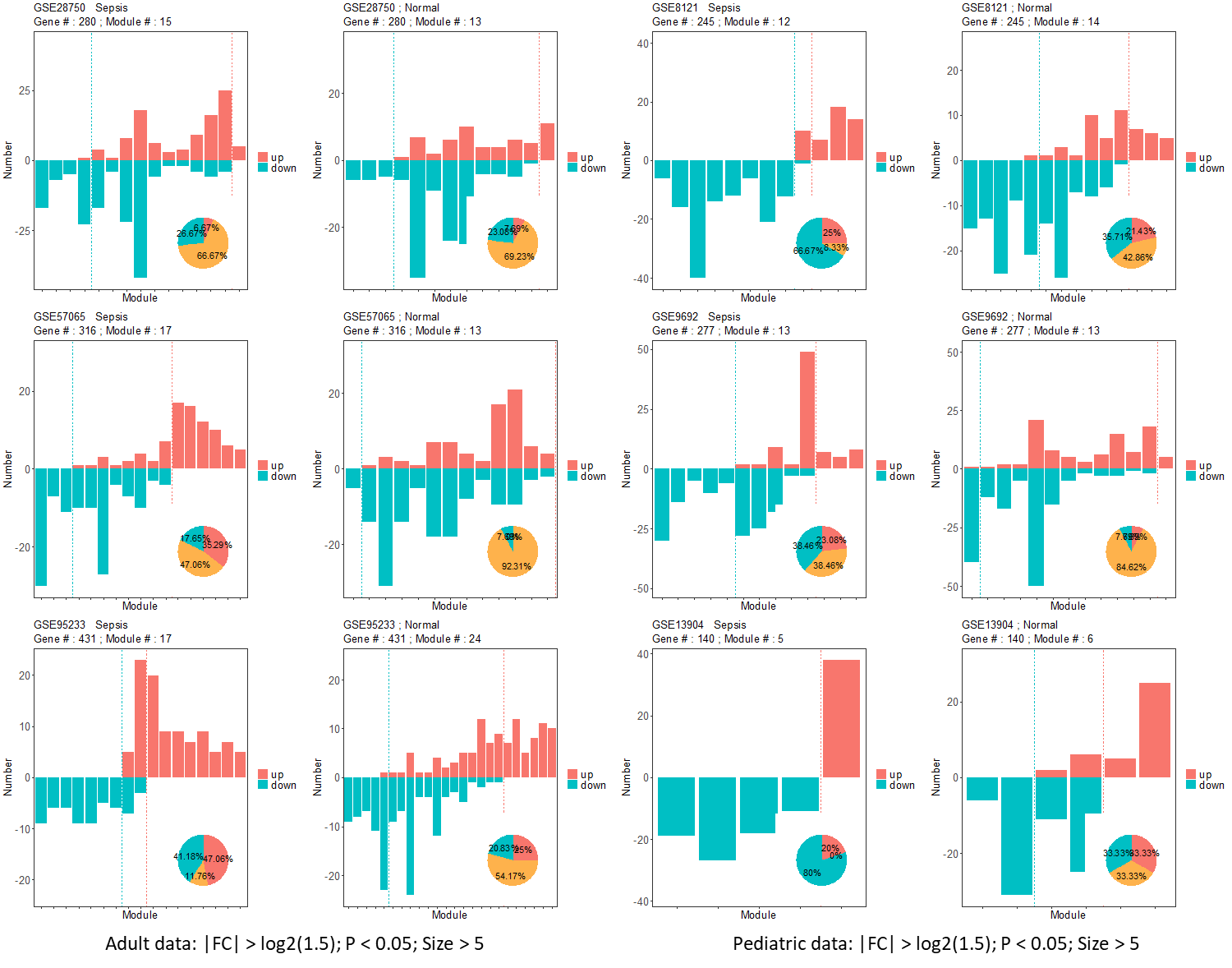
**

**Figure S3.** Composition of lncRNA modules identified from different states. Y axis represents the number of up or down-regulated lncRNAs in each module. Different module types are separated by the dashed lines. The embedded pie shows the proportions of each types of modules.


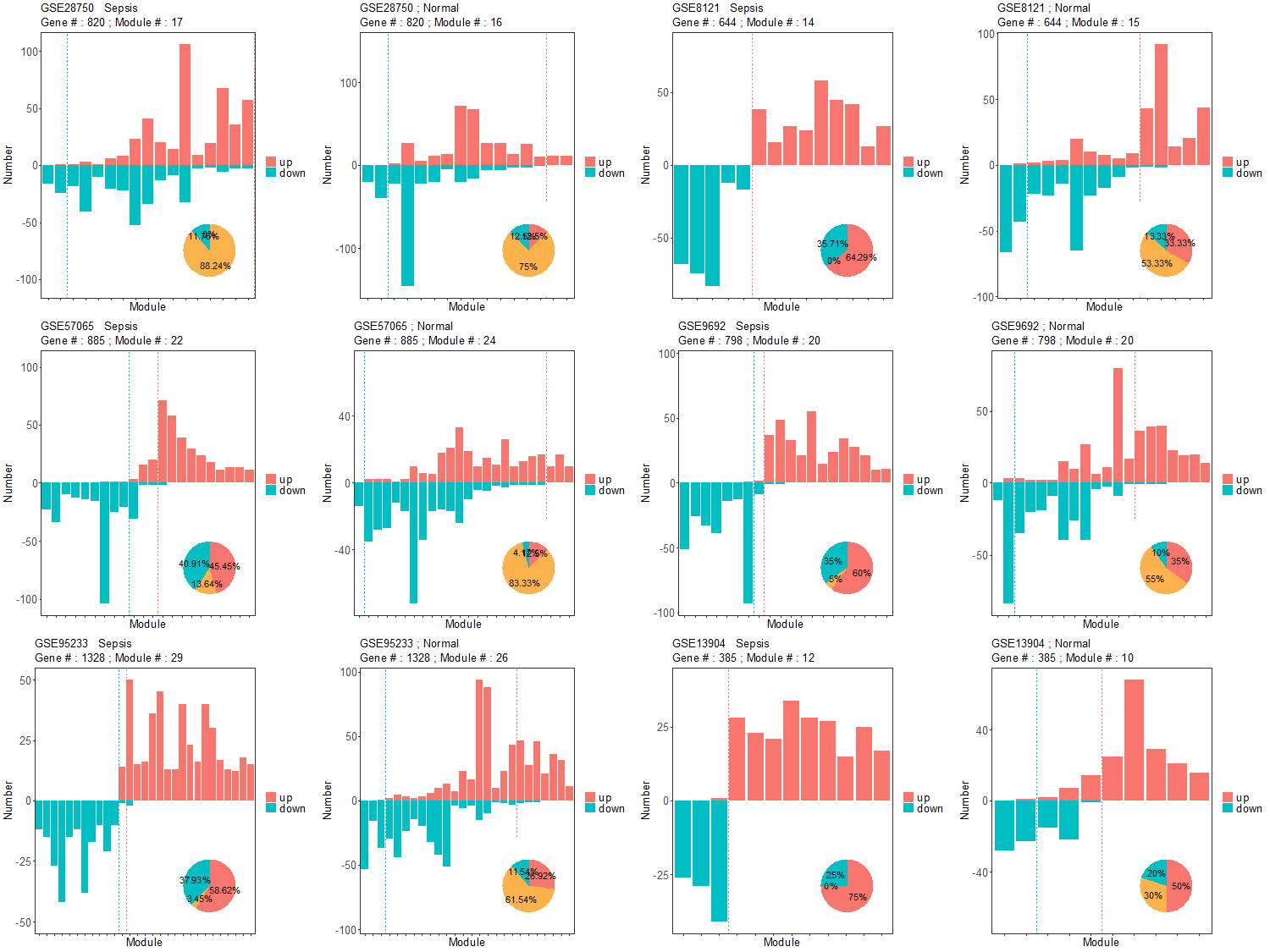
**Figure S4.** The DEGs were screened by the criteria of p value < 0.01 and absolute fold change > 2. The minimum module size is 10.


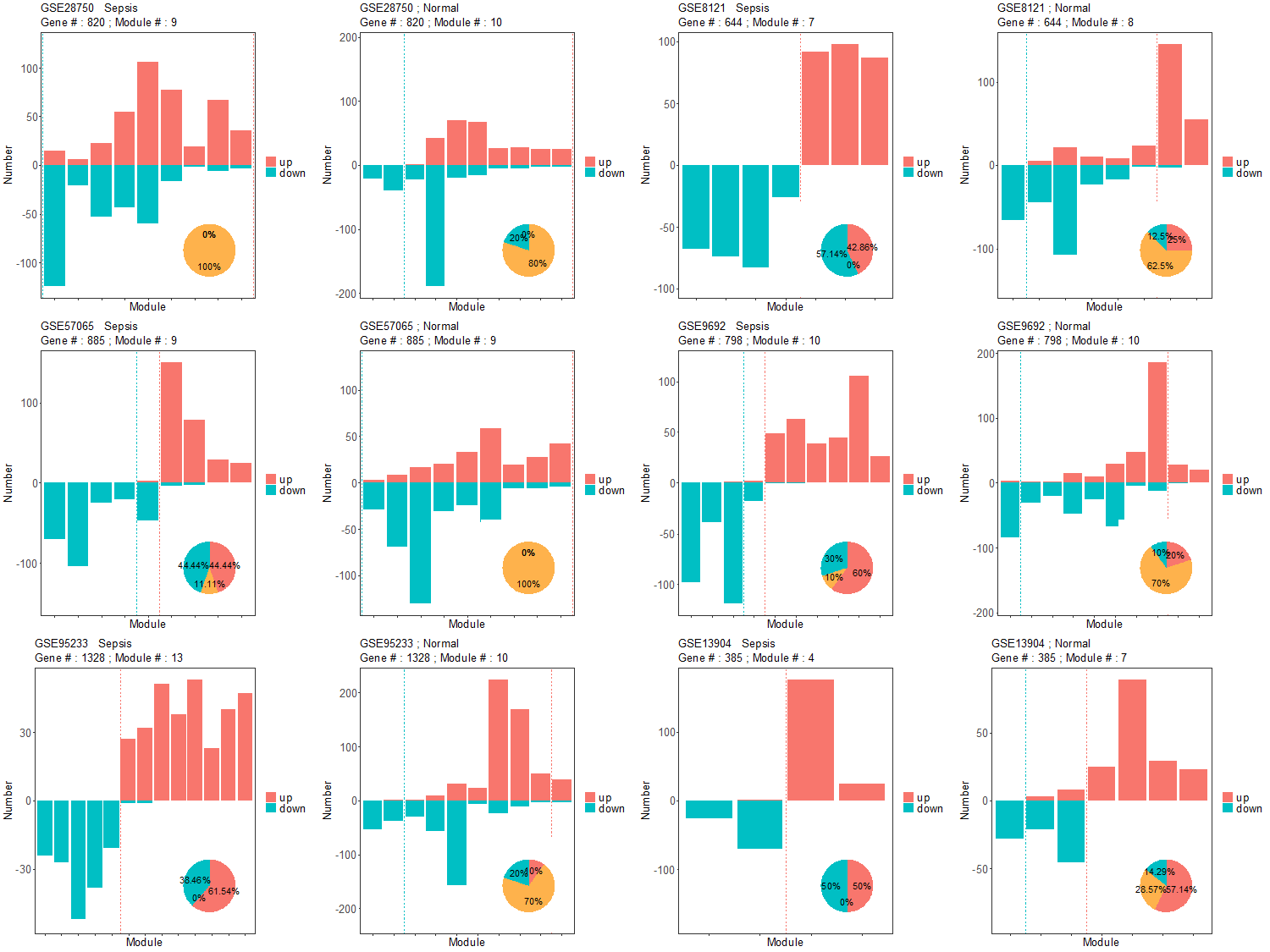


**Figure S5.** The DEGs were screened by the criteria of p value < 0.01 and absolute fold change > 2. The minimum module size is 20.


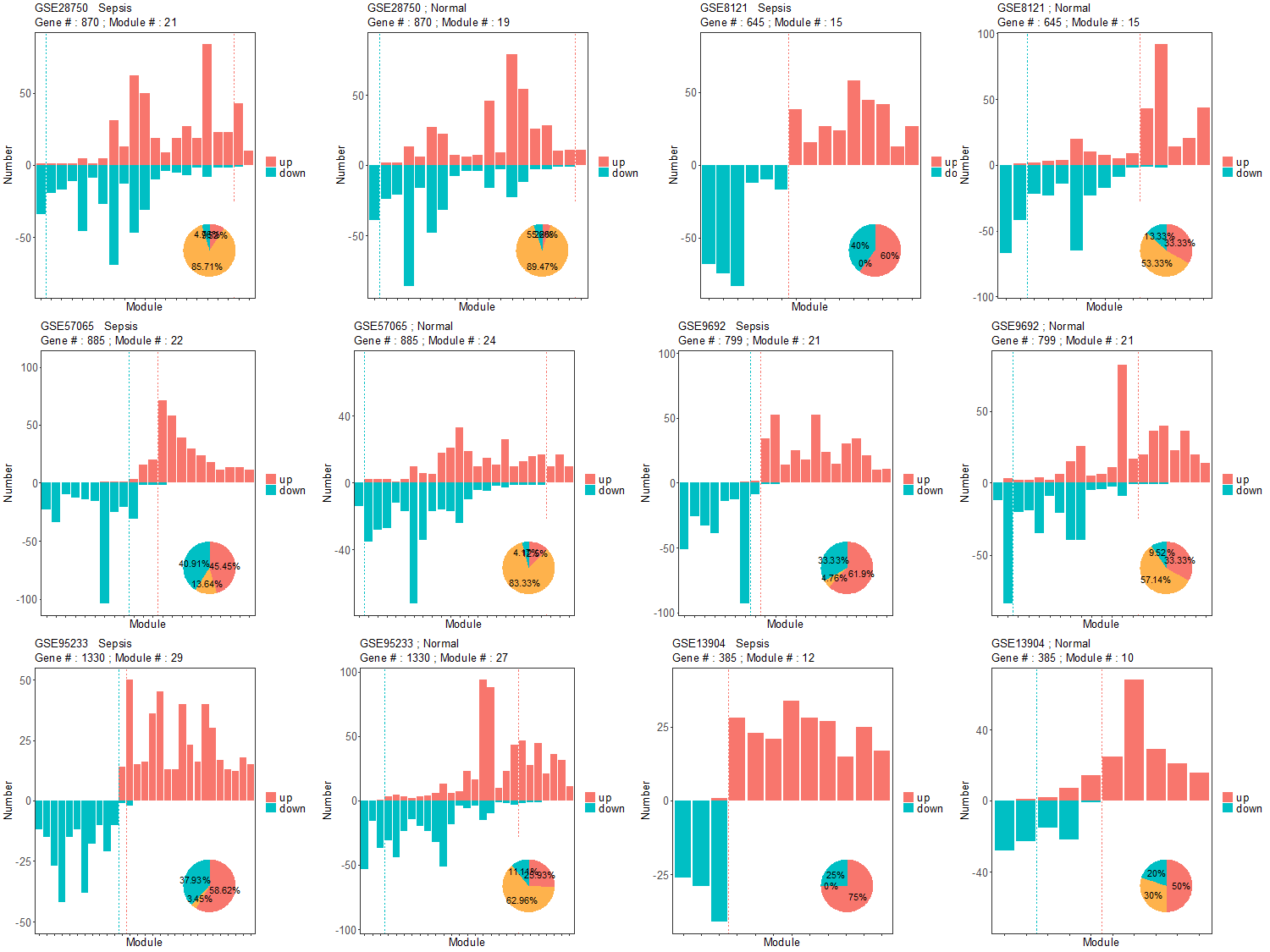


**Figure S6.** The DEGs were screened by the criteria of p value < 0.05 and absolute fold change > 2. The minimum module size is 10.


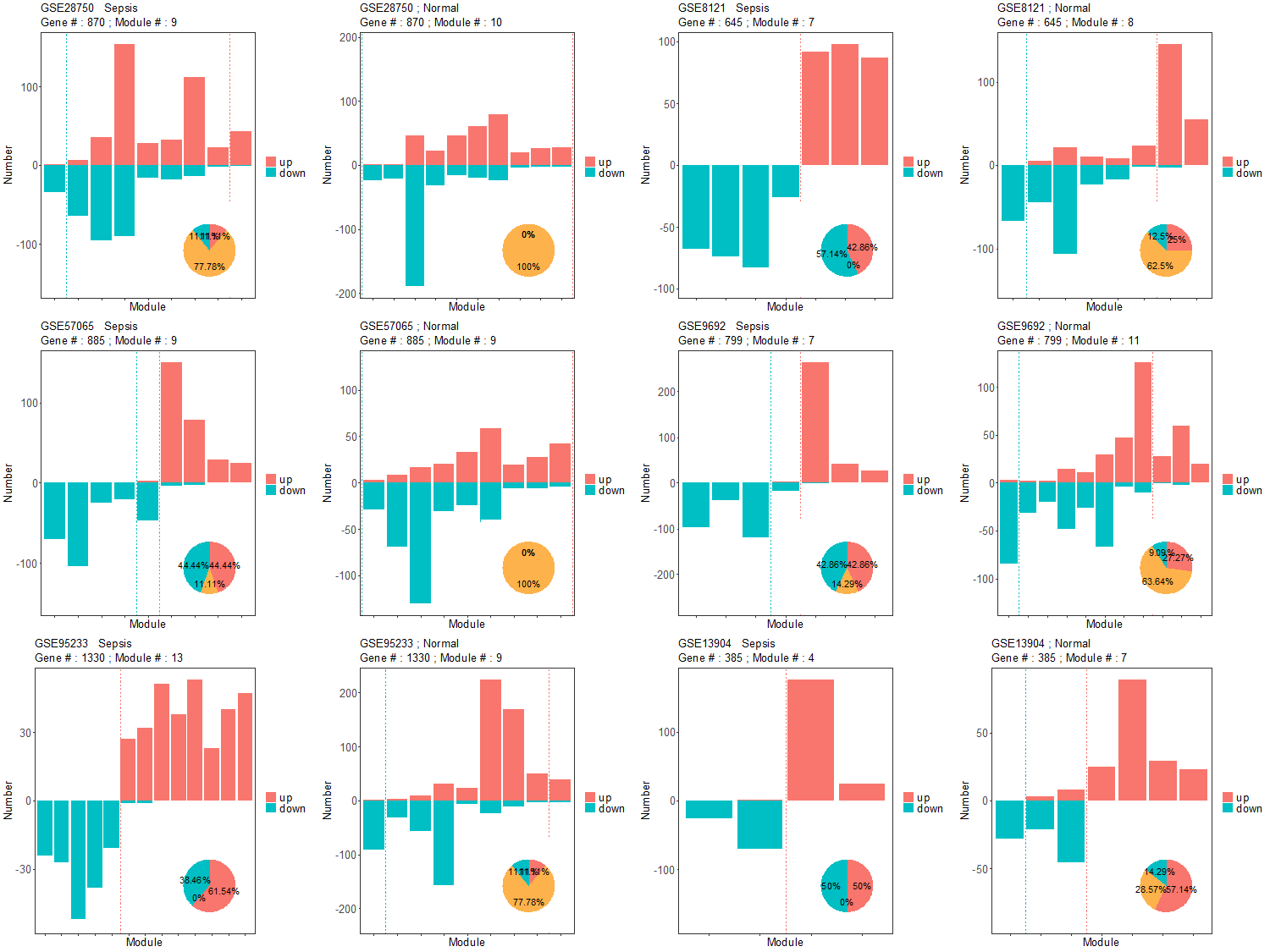


**Figure S7.** The DEGs were screened by the criteria of p value < 0.05 and absolute fold change > 2. The minimum module size is 20.


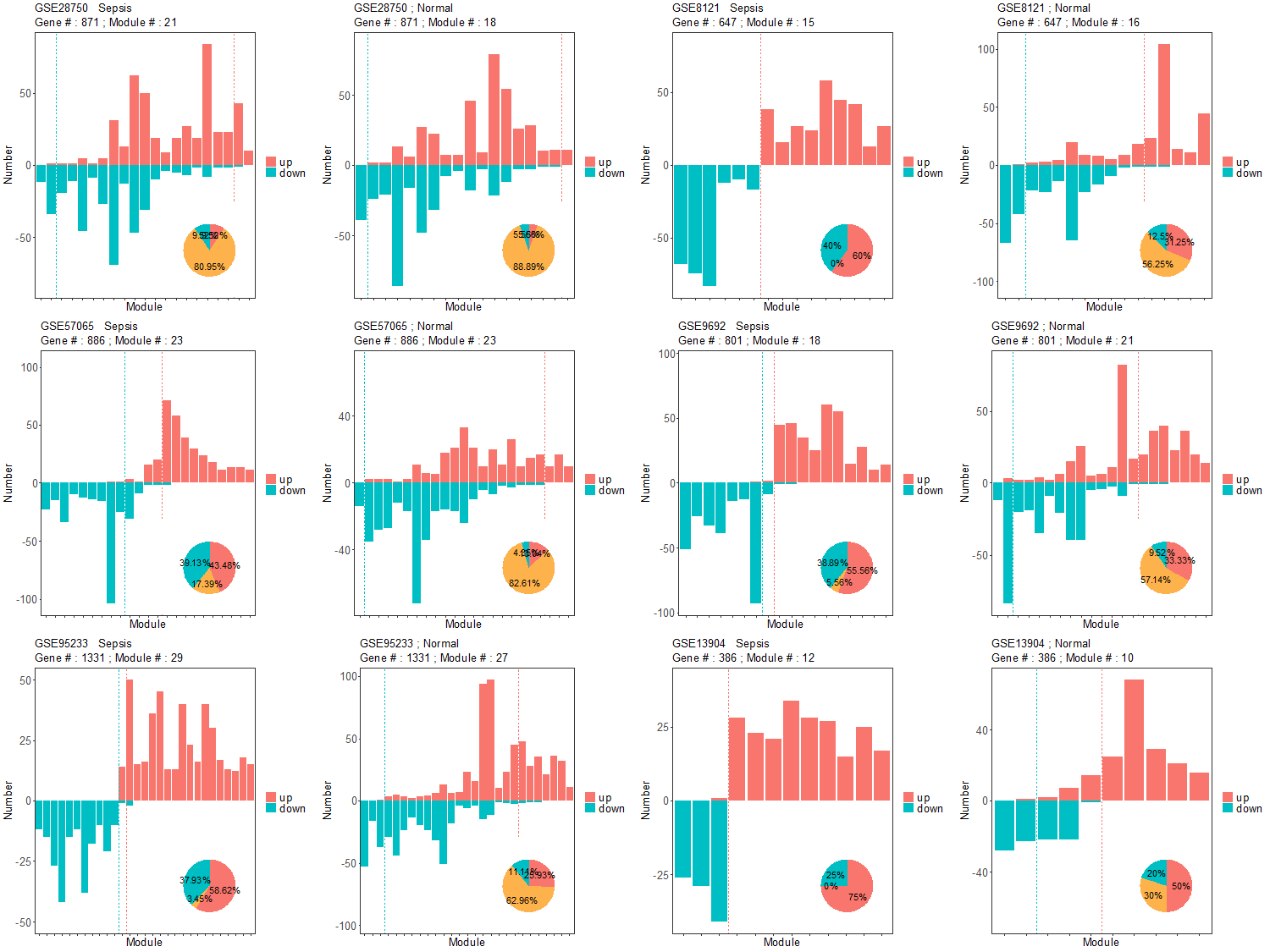


**Figure S8.** The DEGs were screened by the criteria of p value < 1 and absolute fold change > 2. The minimum module size is 10.


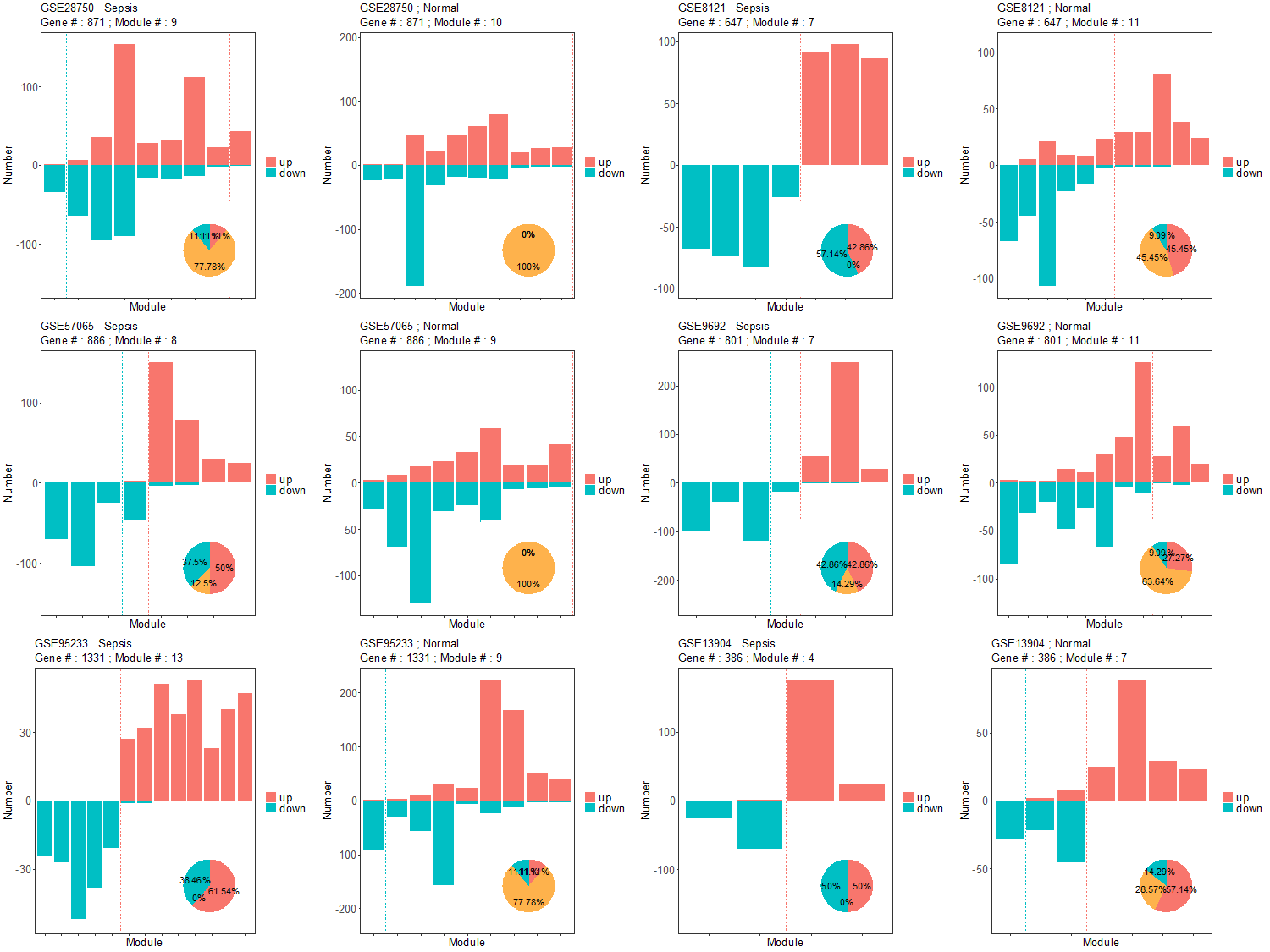


**Figure S9.** The DEGs were screened by the criteria of p value < 1 and absolute fold change > 2. The minimum module size is 20.


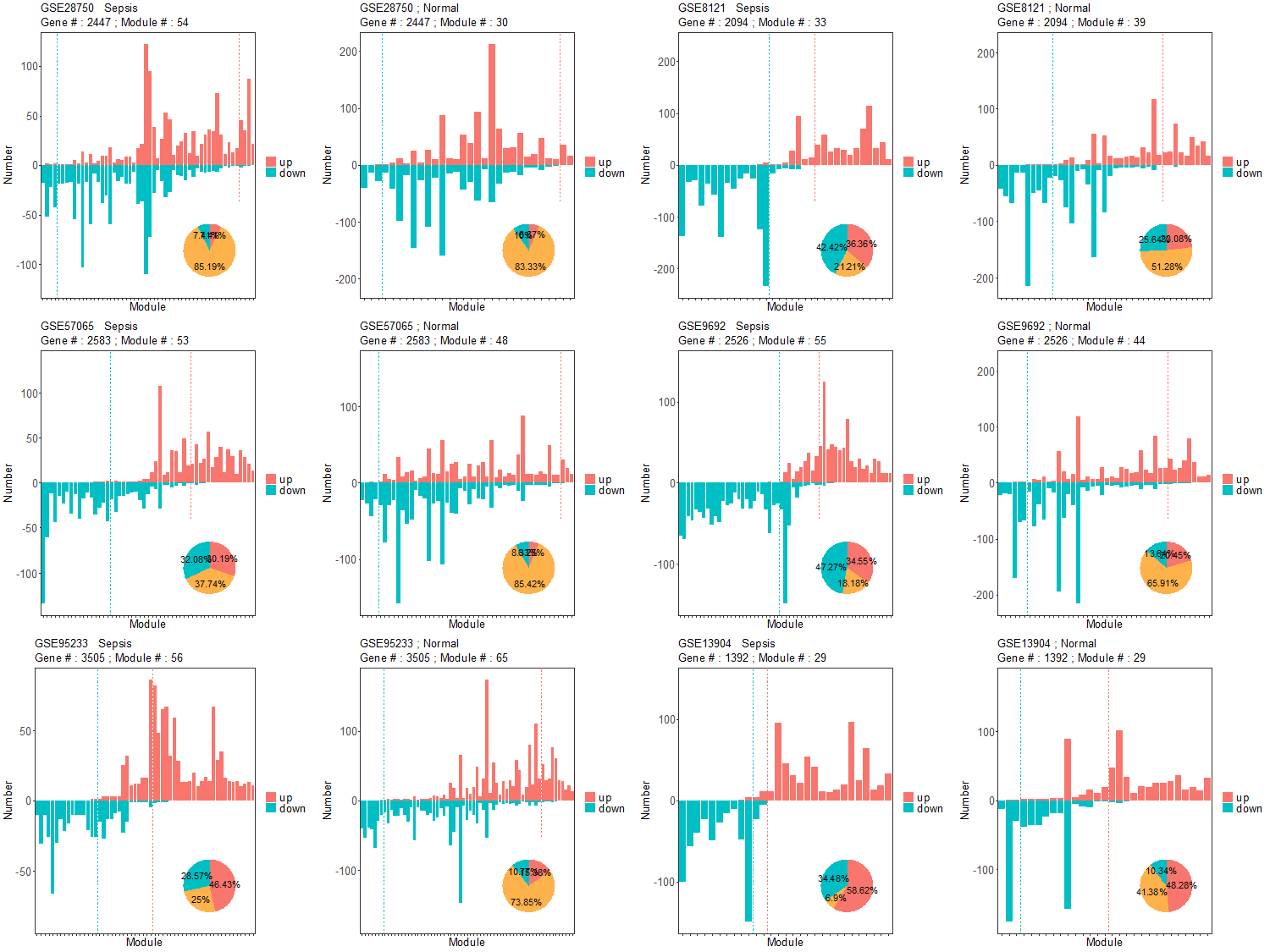


**Figure S10.** The DEGs were screened by the criteria of p value < 1 and absolute fold change > 1.5. The minimum module size is 10.


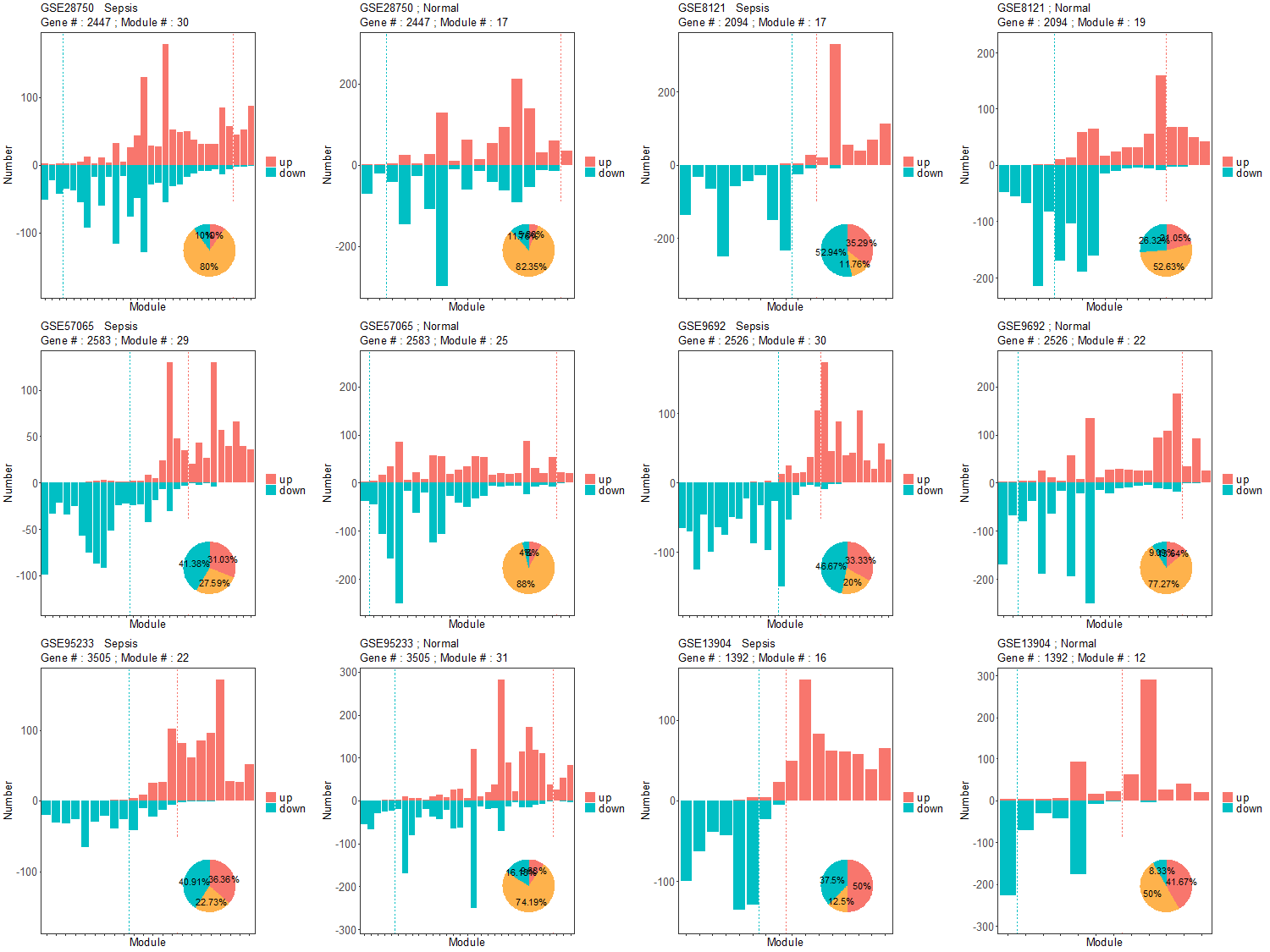


**Figure S11.** The DEGs were screened by the criteria of p value < 1 and absolute fold change > 1.5. The minimum module size is 20.


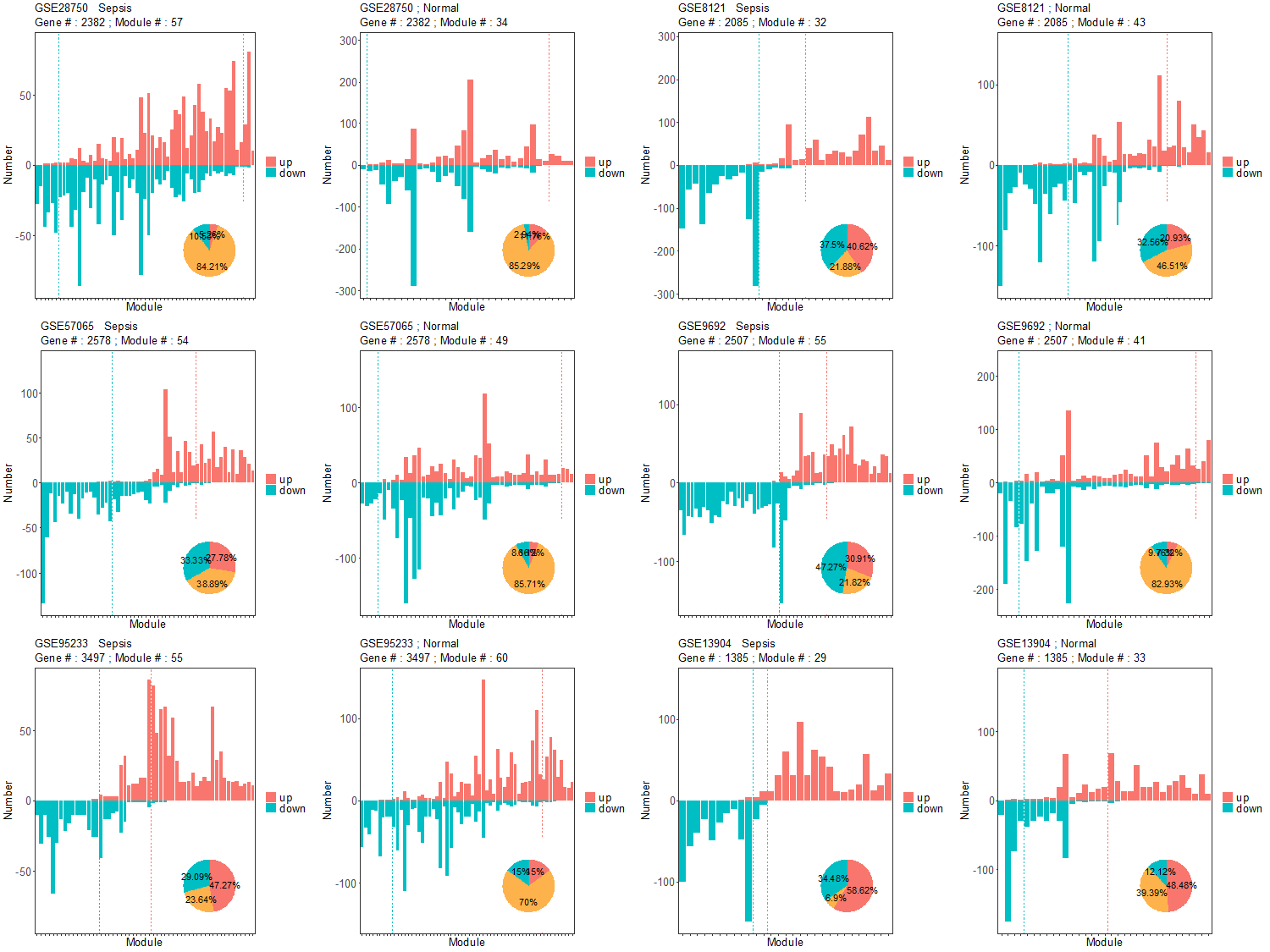


**Figure S12.** The DEGs were screened by the criteria of p value < 0.05 and absolute fold change > 1.5. The minimum module size is 10.


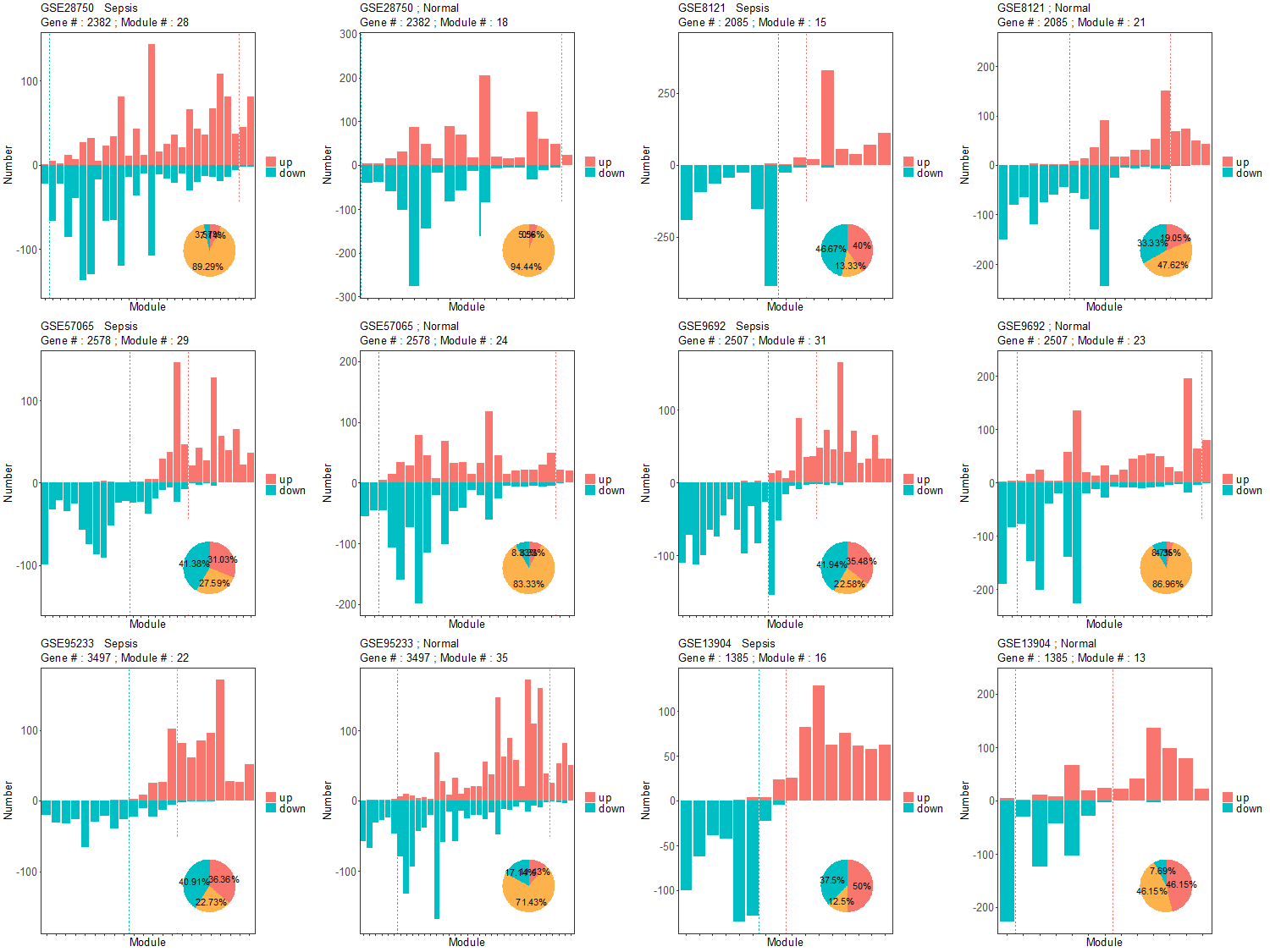


**Figure S13.** The DEGs were screened by the criteria of p value < 0.05 and absolute fold change > 1.5. The minimum module size is 20.


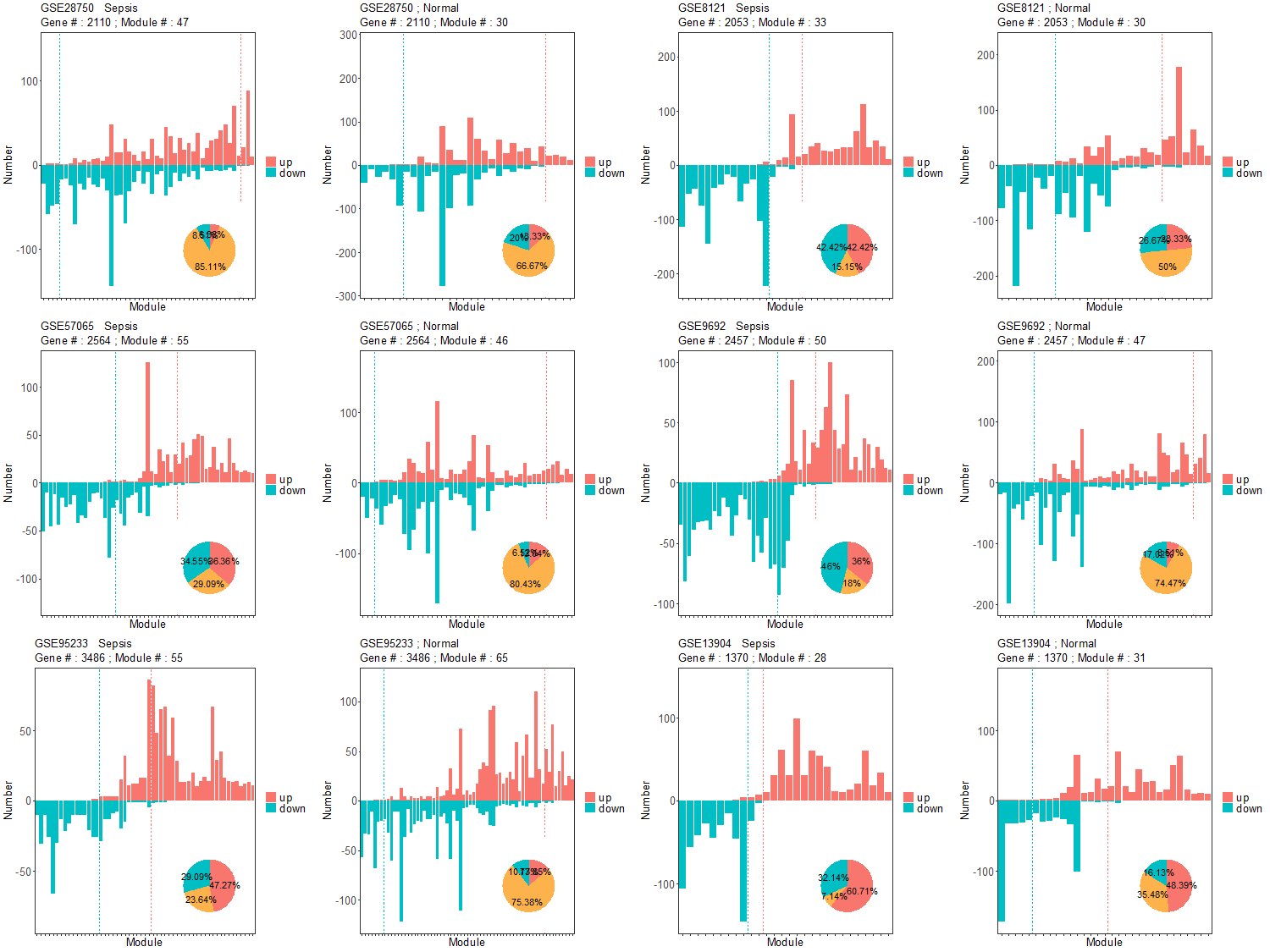


**Figure S14.** The DEGs were screened by the criteria of p value < 0.01 and absolute fold change > 1.5. The minimum module size is 10.


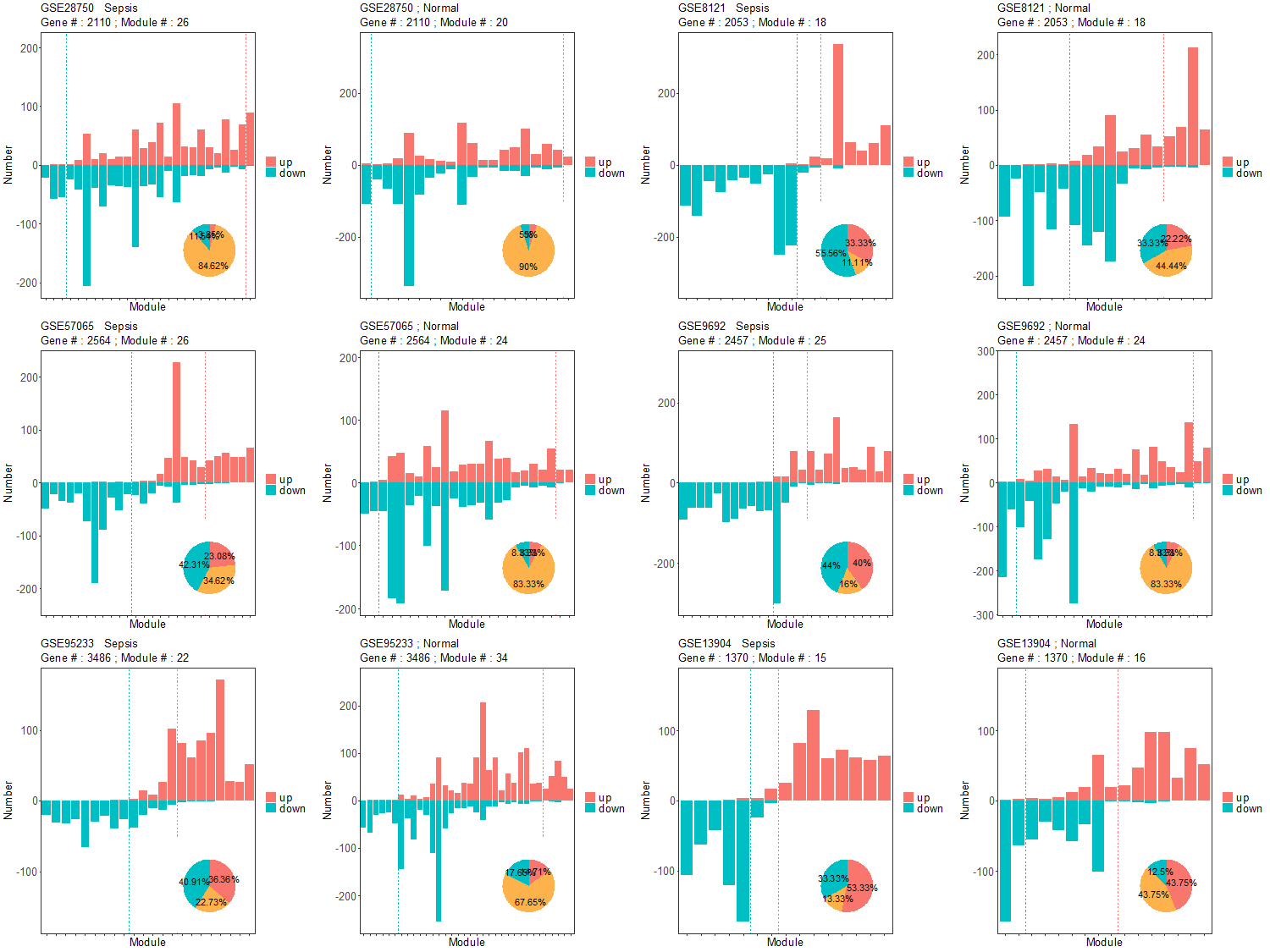


**Figure S15.** The DEGs were screened by the criteria of p value < 0.01 and absolute fold change > 1.5. The minimum module size is 20.


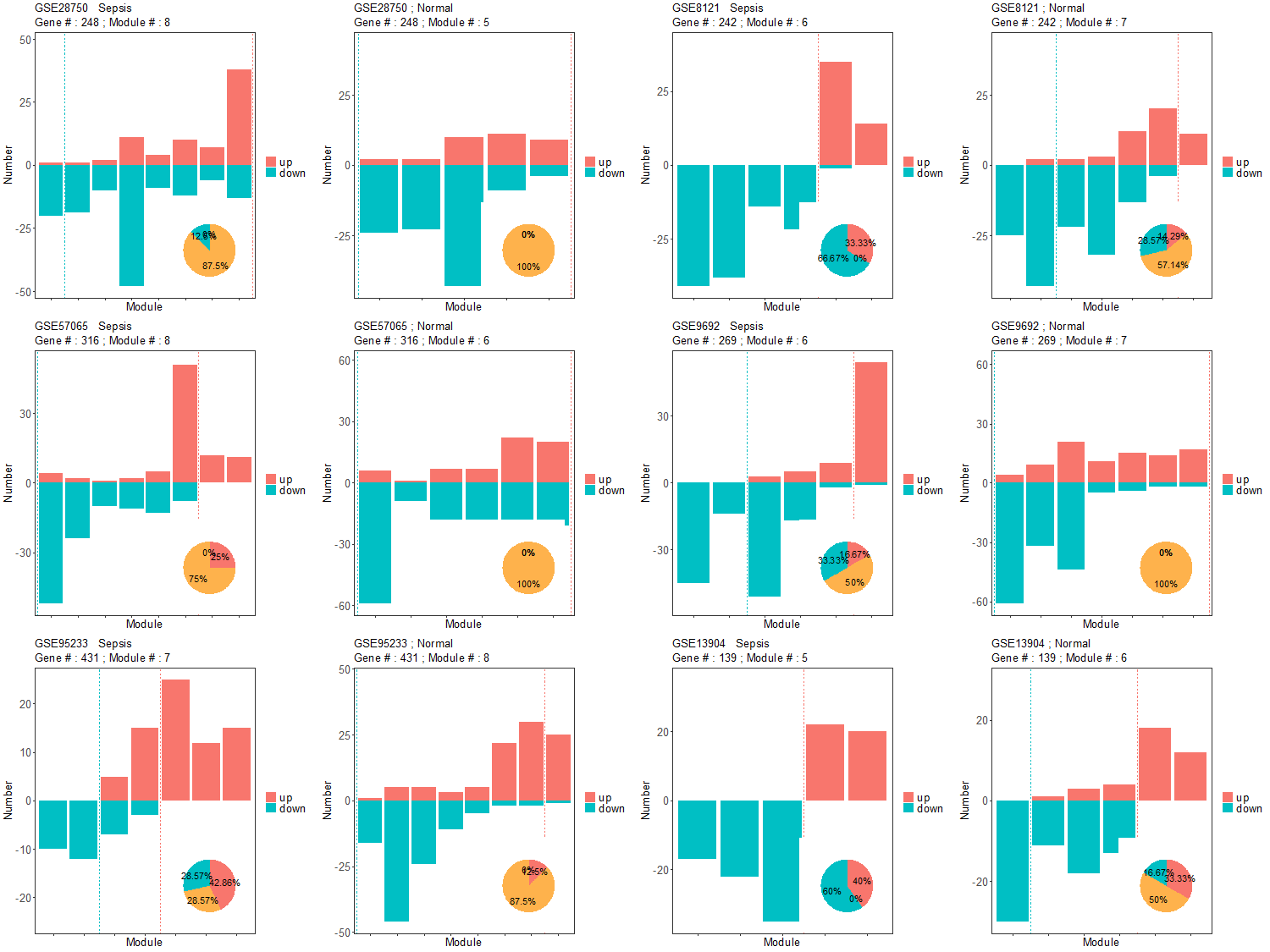


**Figure S16.** The DELs were screened by the criteria of p value < 0.01 and absolute fold change > 1.5. The minimum module size is 10.


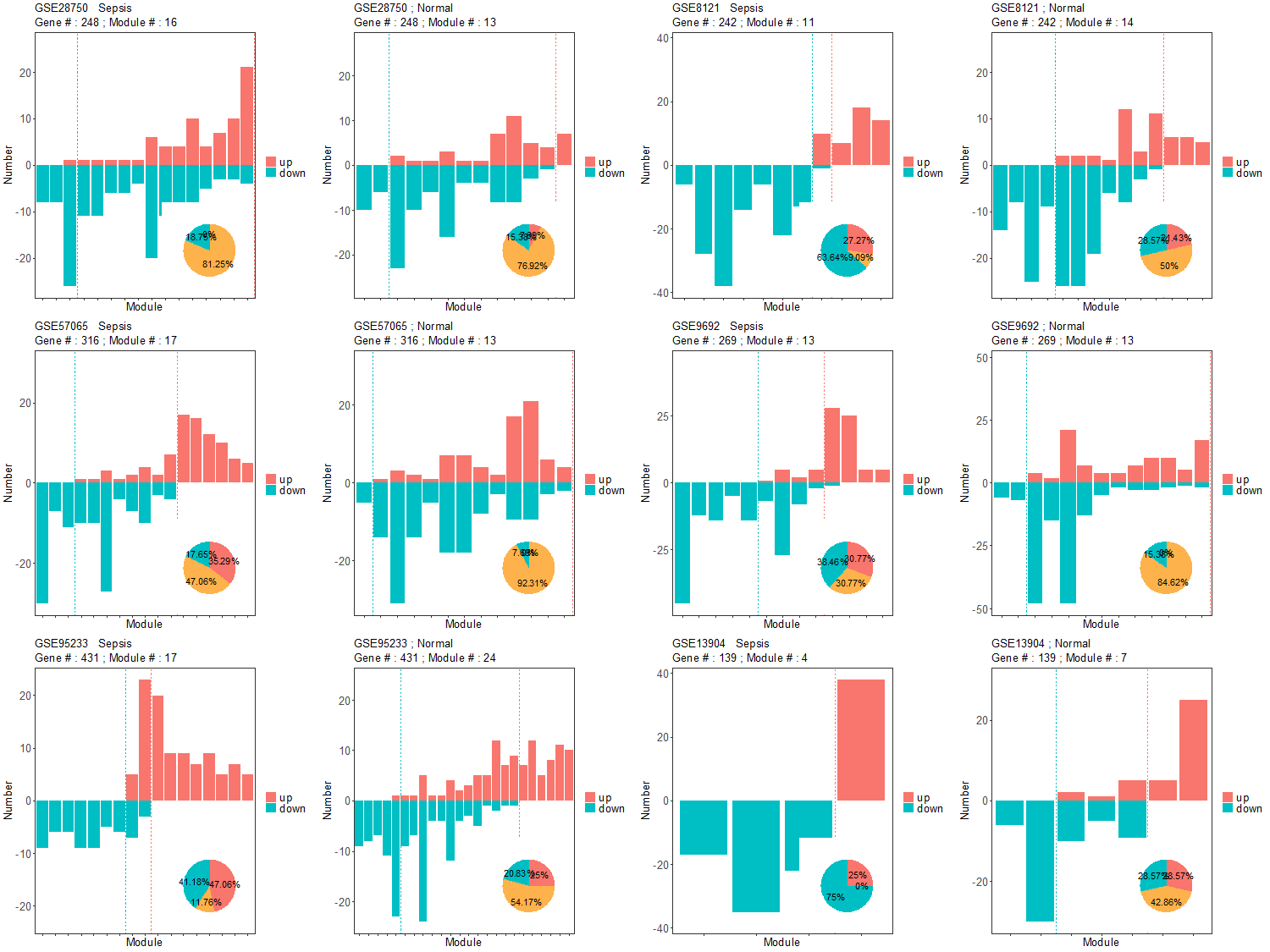


**Figure S17.** The DELs were screened by the criteria of p value < 0.01 and absolute fold change > 1.5. The minimum module size is 5.


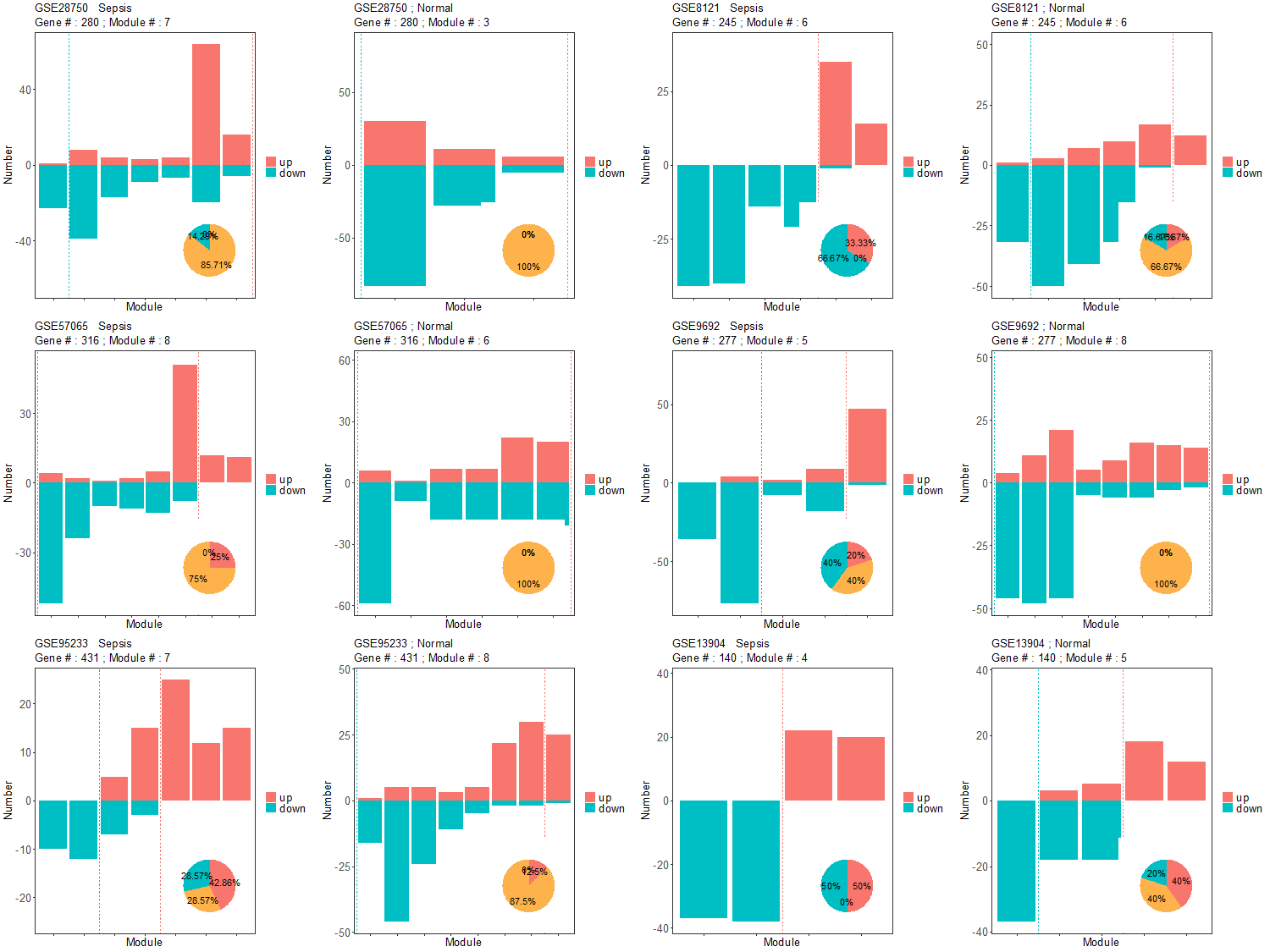


**Figure S18.** The DELs were screened by the criteria of p value < 0.05 and absolute fold change > 1.5. The minimum module size is 10.


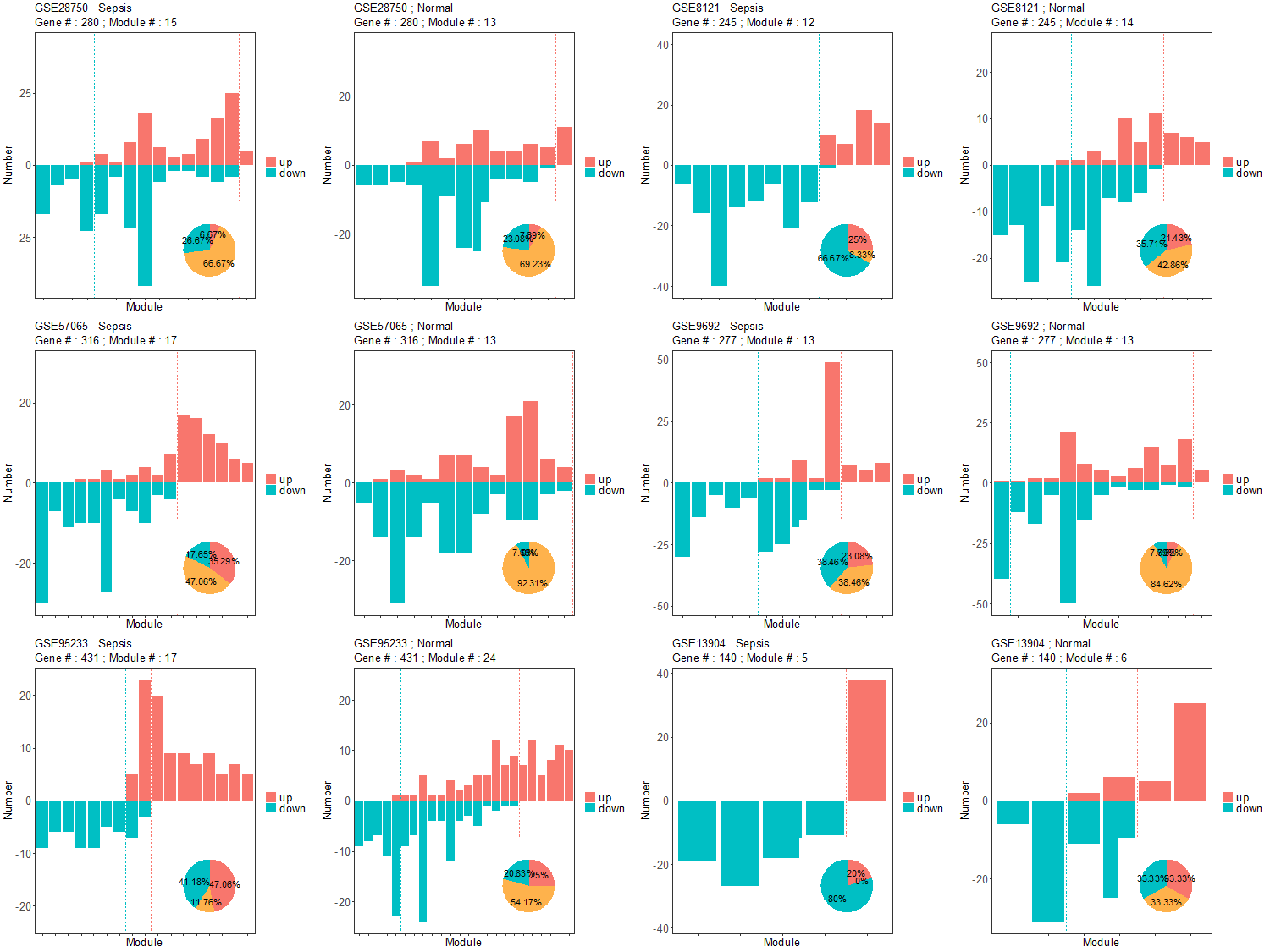


**Figure S19.** The DELs were screened by the criteria of p value < 0.05 and absolute fold change > 1.5. The minimum module size is 5.


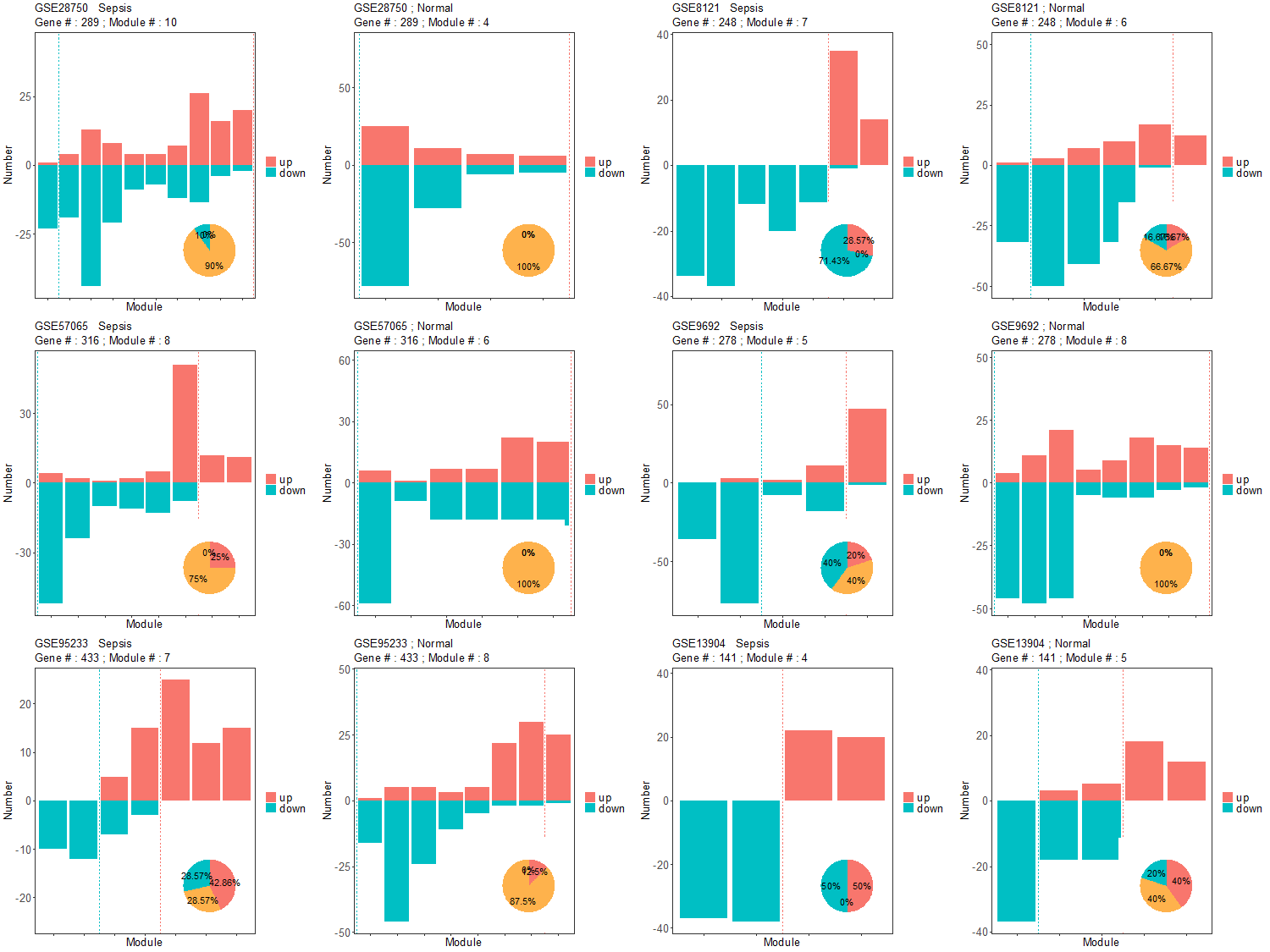


**Figure S20.** The DELs were screened by the criteria of p value < 1 and absolute fold change > 1.5. The minimum module size is 10.


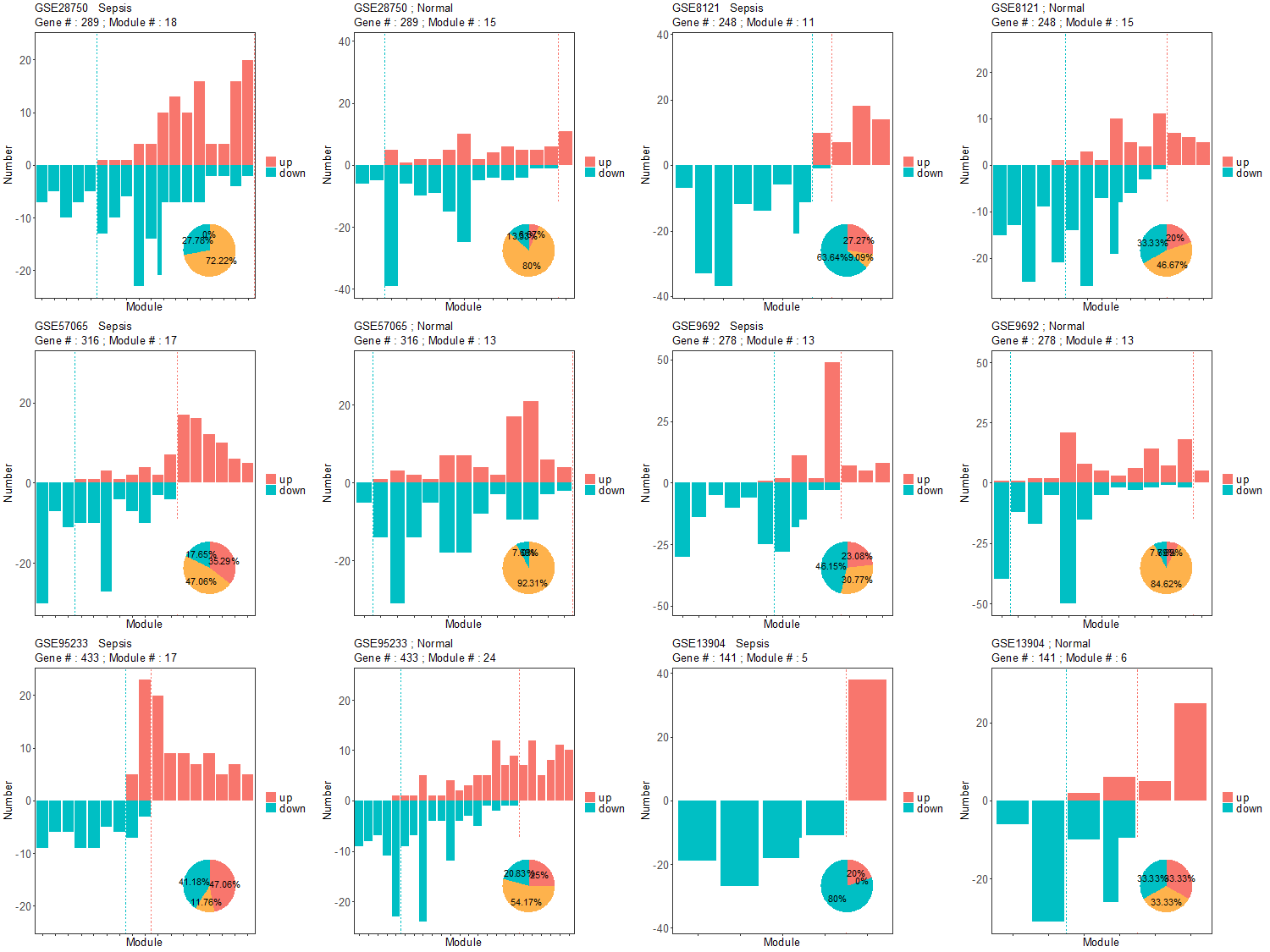


**Figure S21.** The DELs were screened by the criteria of p value < 1 and absolute fold change > 1.5. The minimum module size is 5.


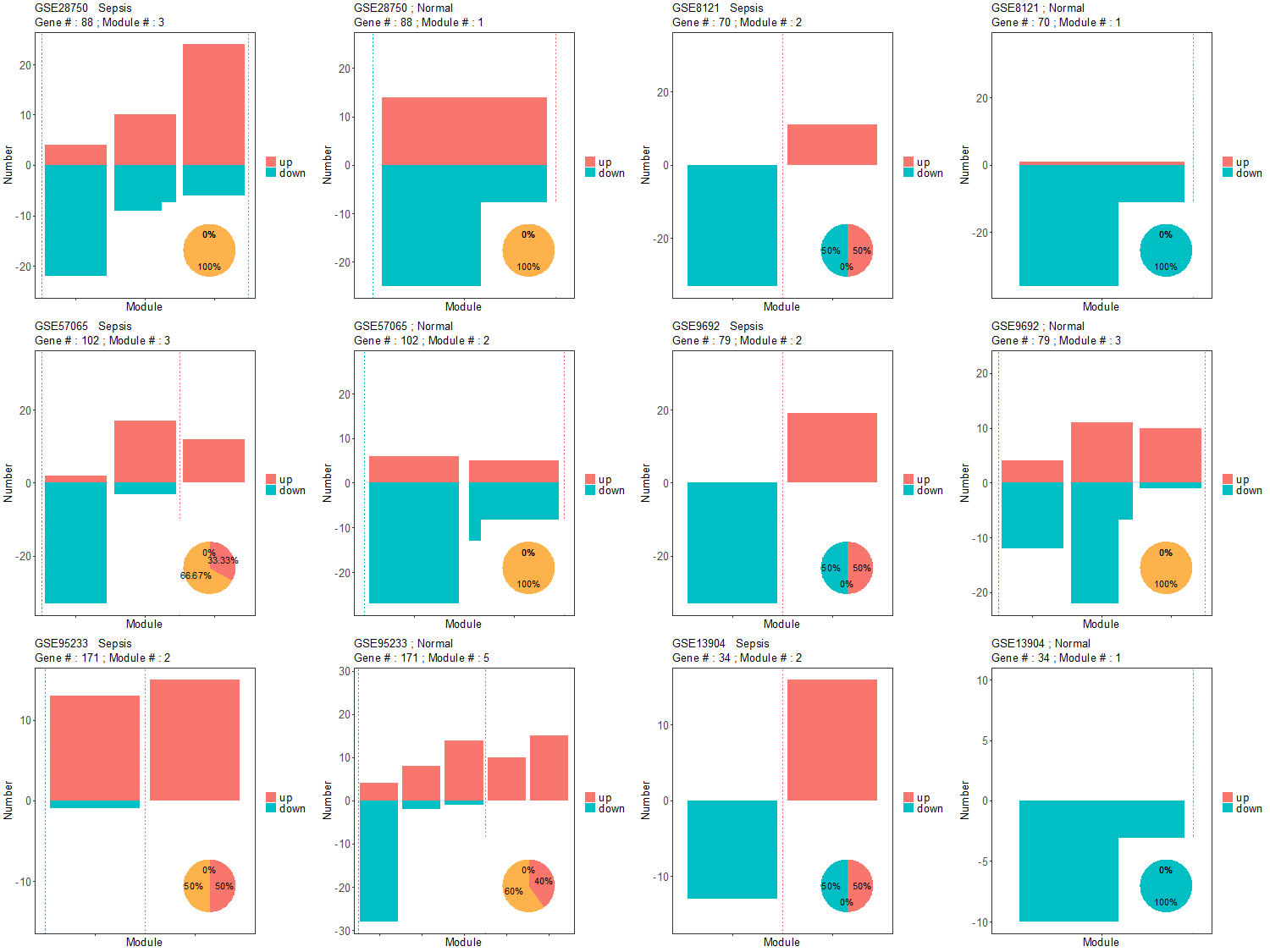


**Figure S22.**. The DELs were screened by the criteria of p value < 0.01 and absolute fold change > 2. The minimum module size is 10.


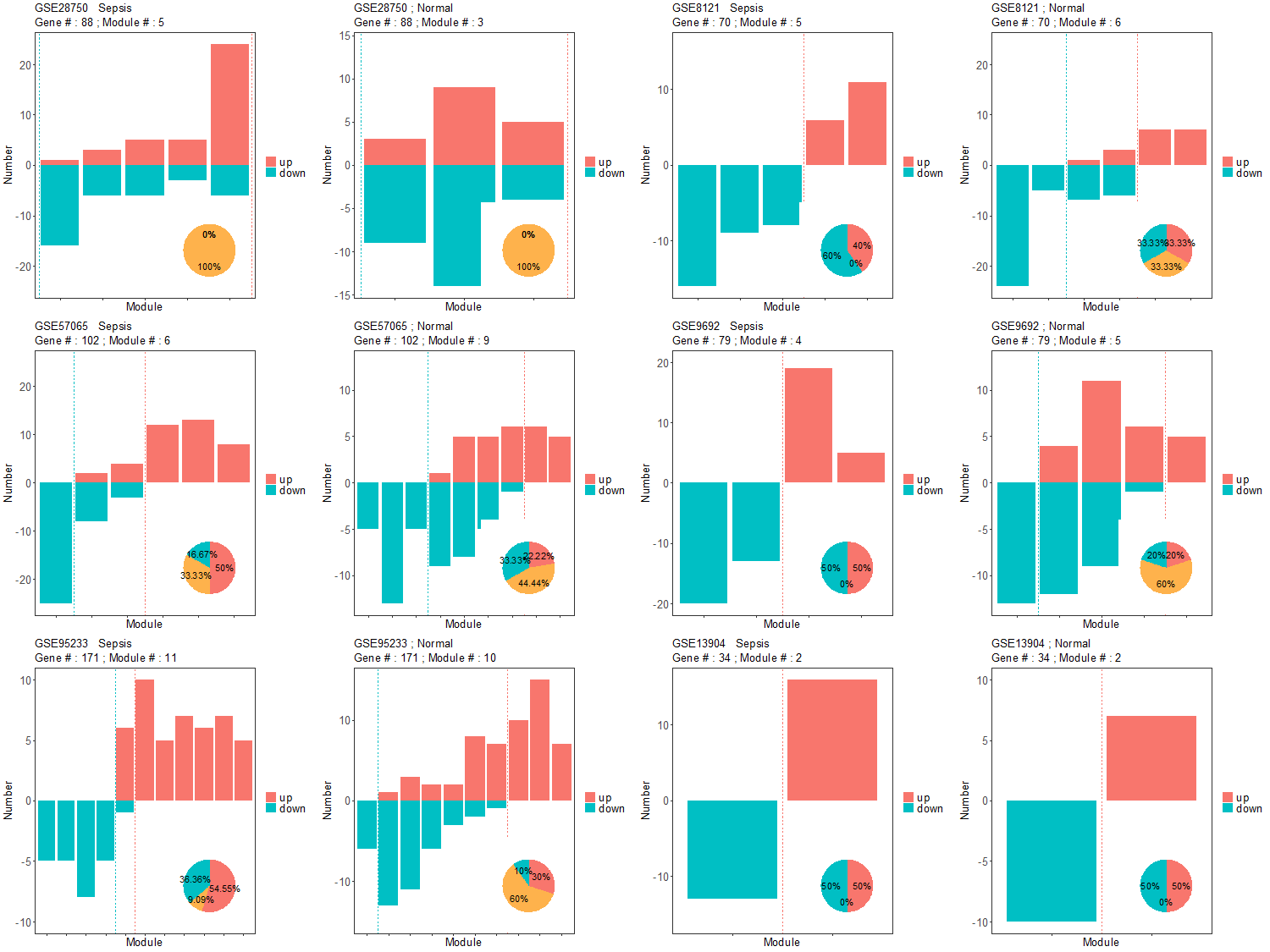


**Figure S23.**. The DELs were screened by the criteria of p value < 0.01 and absolute fold change > 2. The minimum module size is 5.


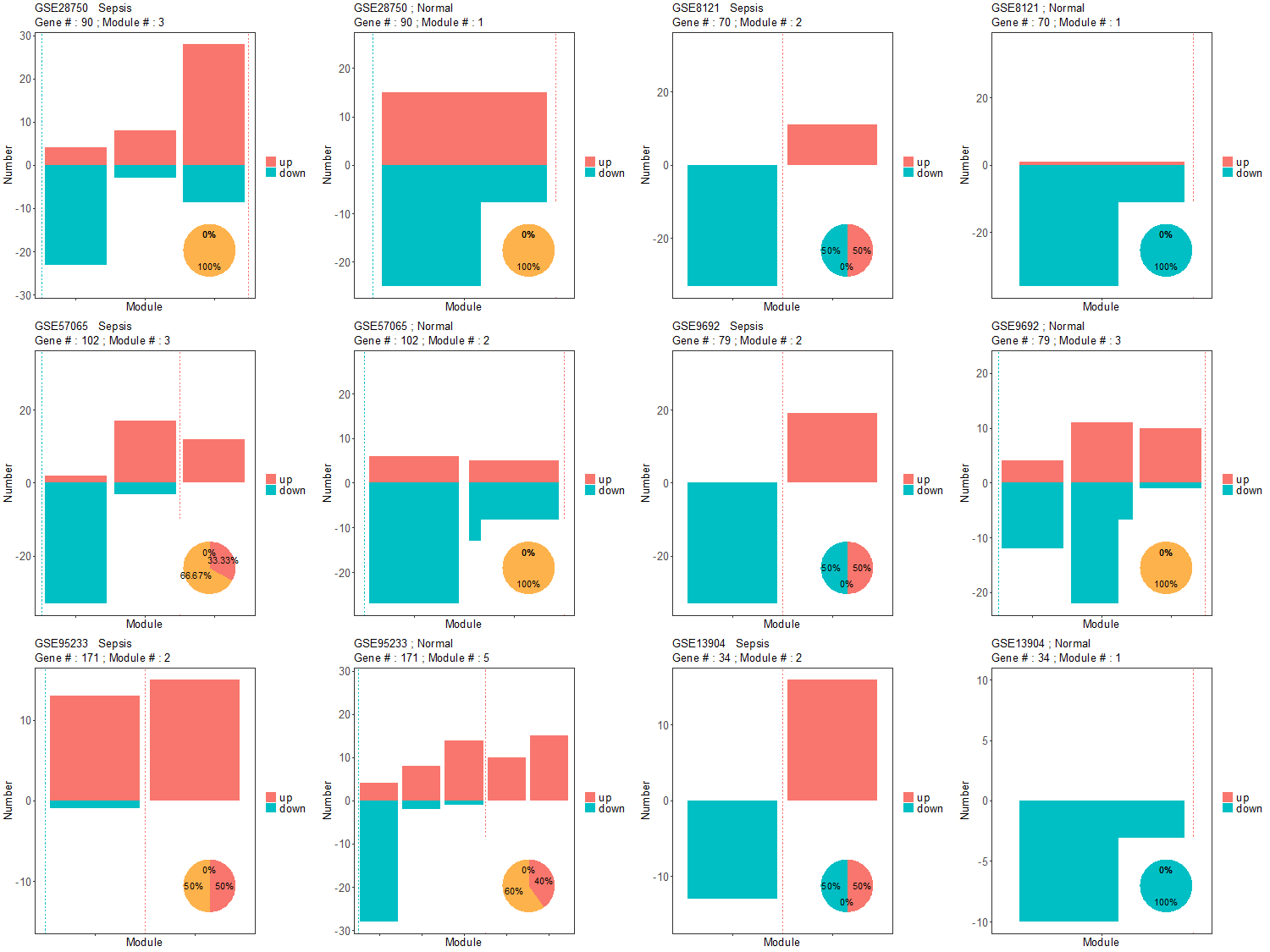


**Figure S24.** The DELs were screened by the criteria of p value < 0.05 and absolute fold change > 2. The minimum module size is 10.


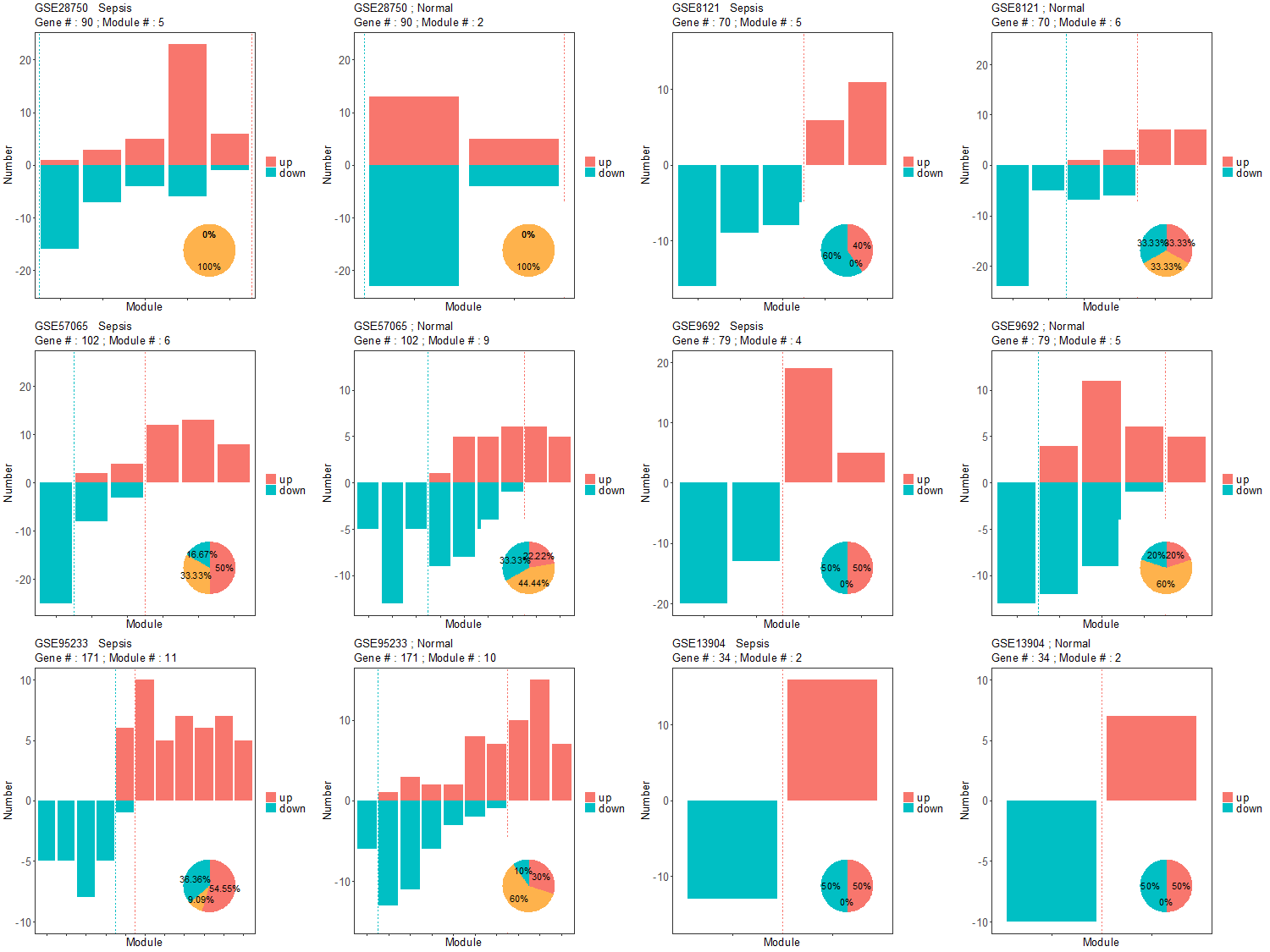


**Figure S25.** The DELs were screened by the criteria of p value < 0.05 and absolute fold change > 2. The minimum module size is 5.


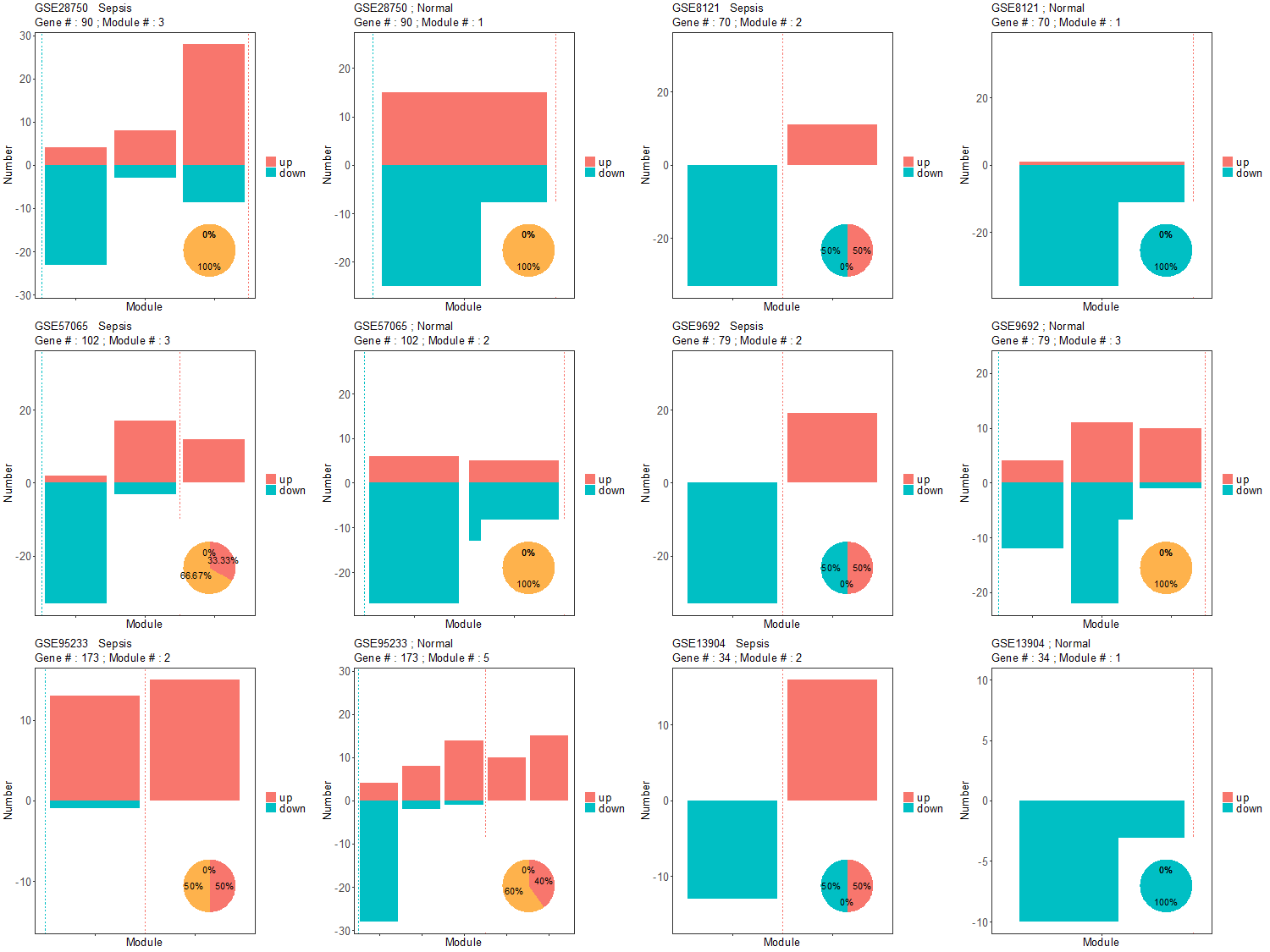


**Figure S26.** The DELs were screened by the criteria of p value < 1 and absolute fold change > 2. The minimum module size is 10.


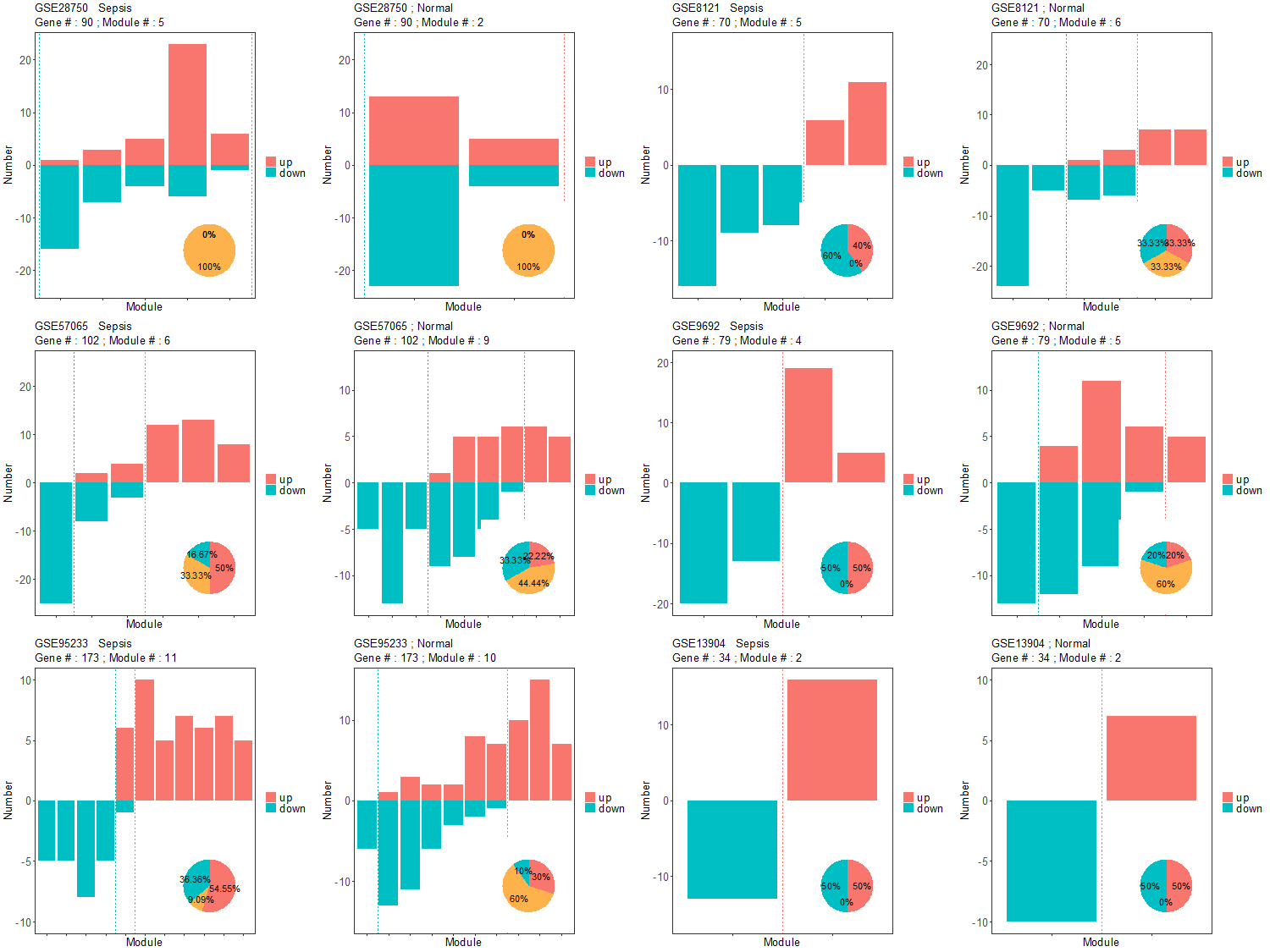


**Figure S27.** The DELs were screened by the criteria of p value < 1 and absolute fold change > 2. The minimum module size is 5.
